# Balloon Angioplasty – The Legacy of Andreas Grüntzig, M.D. (1939–1985)

**DOI:** 10.3389/fcvm.2014.00015

**Published:** 2014-12-29

**Authors:** Matthias Barton, Johannes Grüntzig, Marc Husmann, Josef Rösch

**Affiliations:** ^1^University of Zürich, Zürich, Switzerland; ^2^Heinrich Heine-Universität, Düsseldorf, Germany; ^3^University Hospital Zürich, Zürich, Switzerland; ^4^Dotter Interventional Institute, OHSU, Portland, OR, USA

**Keywords:** Andreas Gruentzig, autobiography, atherosclerosis, Alexis Carrel, biography, coronary artery disease, Robert Hegglin, Nobel Prize, peripheral vascular disease

## Abstract

In 1974, at the Medical Policlinic of the University of Zürich, German-born physician-scientist Andreas Grüntzig (1939–1985) for the first time applied a balloon-tipped catheter to re-open a severely stenosed femoral artery, a procedure, which he initially called “percutaneous transluminal dilatation”. Balloon angioplasty as a therapy of atherosclerotic vascular disease, for which Grüntzig and Charles T. Dotter (1920–1985) received a nomination for the Nobel Prize in Physiology or Medicine in 1978, became one of the most successful examples of translational medicine in the twentieth century. Known today as percutaneous transluminal angioplasty (PTA) in peripheral arteries or percutaneous transluminal coronary angioplasty (PTCA) or percutaneous coronary intervention (PCI) in coronary arteries, balloon angioplasty has become the method of choice to treat patients with acute myocardial infarction or occluded leg arteries. On the occasion of the 40^th^ anniversary of balloon angioplasty, we summarize Grüntzig’s life and career in Germany, Switzerland, and the United States and also review the developments in vascular medicine from the 1890s to the 1980s, including Dotter’s first accidental angioplasty in 1963. The work of pioneers of catheterization, including Pedro L. Fariñas in Cuba, André F. Cournand in France, Werner Forssmann, Werner Porstmann and Eberhard Zeitler in Germany, António Egas Moniz and Reynaldo dos Santos in Portugal, Sven-Ivar Seldinger in Sweden, and Barney Brooks, Thomas J. Fogarty, Melvin P. Judkins, Richard K. Myler, Dickinson W. Richards, and F. Mason Sones in the United States, is discussed. We also present quotes by Grüntzig and excerpts from his unfinished autobiography, statements of Grüntzig’s former colleagues and contemporary witnesses, and have included hitherto unpublished historic photographs and links to archive recordings and historic materials. This year, on June 25, 2014, Andreas Grüntzig would have celebrated his 75^th^ birthday. This article is dedicated to his memory.

## Atherosclerotic Vascular Disease: An ‘Intention to Treat’

Atherosclerosis of arterial blood vessels represents the major cause of illness, disability, and death in most of the world’s industrialized countries, with numbers increasing also in developing countries (1–4). Due to the multifactorial nature of atherosclerotic vascular disease, no specific treatment is available (1, 2, 5), and its prevention and therapy continue to pose great tasks for physicians, healthcare providers, and governments (6). Advances in understanding, diagnosis, and treatment of vascular disease have been made possible through discoveries in pathology (7–9), biochemistry (10), molecular biology (11), genetics (12), pharmacology (13, 14), surgery (15–21), as well as in engineering technology, which resulted in new modalities to restore blood flow in diseased blood vessels (22–25). However, because of the multiple factors causally involved in atherogenesis (1, 5), attempts to deal with this disease had to be based on an ‘intention to treat’ rather than an ‘intention to cure’ (5).

In the early 1970s, German-born physician-scientist Andreas Grüntzig, M.D. (1939–1985) by inventing balloon angioplasty at the Medical Policlinic of the University of Zürich made one of the most important technological and therapeutic advances in medicine of the twentieth century. Within only a few years, Grüntzig succeeded in developing an idea into a functional technical device, which allowed for the first time to expand a stenosed artery from the lumen by applying pressure to a balloon located at the catheter tip. First introduced in 1974 (22, 26), balloon angioplasty has since become the most frequently used treatment in vascular emergencies of the heart and the peripheral circulation. Grüntzig’s discovery mainly initiated the « Phase 3 » of treatment of acute myocardial infarction as recently defined by Eugene Braunwald, M.D. (« myocardial reperfusion », 1975-present) (27), saving patients from death and disability. On the occasion of the 40^th^ anniversary of Grüntzig’s invention, we will review the historical developments in medicine that led the way toward intraluminal therapy and provide an overview of the stages of Grüntzig’s life that were central in the development of his new method.

## Andreas Grüntzig: Pre-War Germany, Argentina, and East Germany

**Figure 1:**
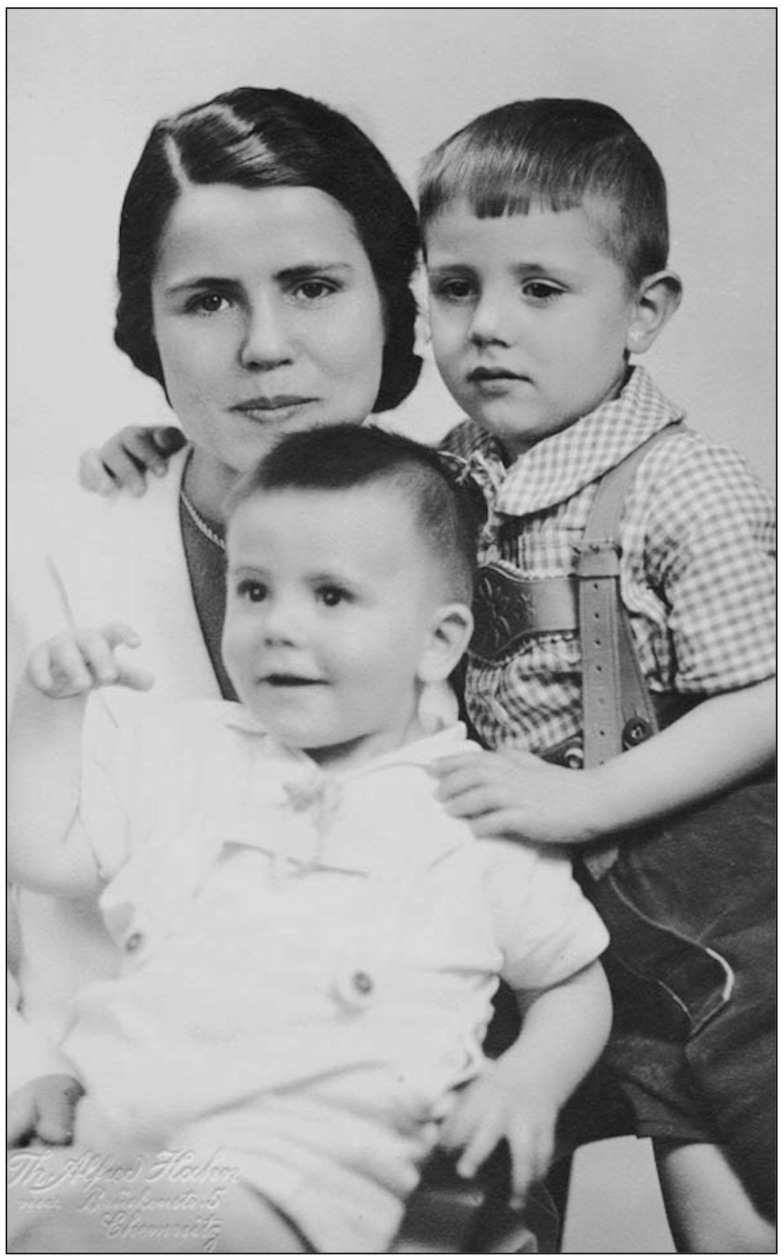
**Portrait of Charlotte Grüntzig with Andreas (sitting) and Johannes Grüntzig (right)**. The photograph is embossed by a mark of Photostudio Th. Alfred Hahn in Chemnitz, Germany, where the photograph was taken in 1942. Photograph reproduced with permission of Johannes Grüntzig, M.D., Düsseldorf.

Andreas Grüntzig was born on a Sunday, June 25, 1939, in Dresden, Germany (28), shortly before the Second World War. His parents were Dr. Willmar Grüntzig (1902–1945), a chemist who also had studied medicine, and Charlotte Grüntzig, née Zeugner (1907–1995), a teacher. Shortly after Grüntzig was born, the family moved to Rochlitz, a small historical town located between Dresden, Leipzig, and Chemnitz (Figure 1). Grüntzig’s father, who served as a meteorologist during the war, went missing just before the war ended. Charlotte Grüntzig and her two sons left for Argentina in 1950 to live with their relatives, but returned to Leipzig, now East Germany, in 1951 (28) because Charlotte Grüntzig insisted that an excellent education was of utmost priority for her children (28). Andreas Grüntzig and his brother Johannes entered high school at the Thomasschule zu Leipzig (28), the oldest public classical high school in Germany founded in 1212. In the early eighteenth century, Johann-Sebastian Bach was the St. Thomas Cantor and his children were pupils at the school, which gained world-wide accolades for its Thomanerchor (St. Thomas Boys Choir). Andreas Grüntzig graduated from the Thomasschule in 1957 with highest honors. In 1958, with the intention to become a physician, Grüntzig escaped the country before the Communist DDR government closed East Germany’s borders, to join his brother Johannes at the University of Heidelberg, leaving his mother and grandmother behind in East Germany (28).

## Developments toward Endovascular Therapy between the 1890s and the 1970s

The concept of repairing arterial blood vessels, including the insertion of non-biological structures into the arterial lumen goes back more than 100 years and was first applied by the French surgeon and biologist Alexis Carrel, M.D. (1873–1944) (15, 18, 24). Carrel was awarded the Nobel Prize in Physiology or Medicine in 1912 for his work in vascular medicine, which he started as a young medical student (18). In 1894, when French President Sadi Carnot, a physicist, was assassinated by stabbing with a knife in the abdomen, bleeding to death, Carrel argued that President Carnot could have been saved if surgeons had been able to repair injured blood vessels (18). Andreas Grüntzig’s intention and approach were similar to those of Carrel, namely to repair injured blood vessels. However, for Grüntzig’s approach injury originated from inside the vessel, and it was the luminal obstruction caused by atherosclerotic plaque that he intended to repair (29).

While surgical revascularization or amputation was available for peripheral artery disease, there were no routine options for patients with advanced coronary artery disease until May 9, 1967, when René Favaloro, M.D. (1923–2000) introduced aorto-coronary bypass surgery (19–21, 25). In the 1950s, surgical coronary endarterectomy («currettement») had been introduced. In 1958, the Swedish surgeon Åke Senning, M.D. (1915–2000), further refined this method, using venous patches to seal coronary arteries after removing coronary plaques by endarterectomy (30, 31). Coronary endarterectomy, however, did not become a standard treatment because “intimal stripping” was often associated with complications, some of which also related to the surgical instruments used (32–35). Also in 1958, Senning invented the first cardiac pacemaker (36–38). In 1961, Senning accepted the Chair of Surgery at the University of Zurich and created the first intensive care unit on the European continent (38); he was going to play an essential role for Grüntzig’s success in the 1970s (36–39).

The first angiography of human arteries was performed on January 17, 1896 by the physicist Eduard Haschek, Ph.D. (1875–1947) and the physician Otto Th. Lindenthal, M.D (1872–1947) at the Physics Institute of the University of Vienna (40). The anatomist Julius Tandler, M.D. (1869–1936) had placed the hand of a corpse at their disposal. The arterial vessels were filled with Teichmann’s solution (iodine solution), a mixture of chalk, cinnabar, and paraffin, and applying X-rays [“*X-Strahlen*”] with an exposure time of 57 min resulted in the first angiogram (40). Angiography of peripheral arteries was introduced in 1924 in the United States by Barney Brooks, M.D. (1884–1952) (41) and in Portugal in 1927 by the neurologist António Egas Moniz, M.D. (1875–1955) (42) who in 1949 received the Nobel Prize in Physiology or Medicine. In 1929, his surgeon colleague, Reynaldo dos Santos, M.D. (1880–1970), introduced arteriography (43) and aortography (44).

Catheterization in humans was first performed by the German physician Werner Forssmann, M.D. (1904–1979). In the late summer of 1929, as a surgical resident at the age of 25 years and only one year out of medical school, Forssmann performed right heart catheterization on himself using a urinary catheter at a small provincial hospital at Eberswalde (45). He subsequently lost his job because he had disobeyed orders of the Department Chair (45–47). For these most daring self-experiments, published by Forssmann between 1929 and 1931 (45, 48, 49), he was awarded the Nobel Prize in Medicine in Physiology or Medicine in 1956 having pioneered cardiac catheterization (50). Forssmann shared the prize with the French physician André F. Cournand, M.D. (1885–1988) and his American colleague Dickinson W. Richards, M.D. (1985–1973) (47, 50).

Roughly a decade after Forssmann had performed the first human cardiac catheterization (Andreas Grüntzig had just been born), the Cuban radiologist Pedro L. Fariñas, M.D. (1892–1951) developed a catheter-based technique for arteriography of the aorta by introducing an urethral catheter through an exposed femoral artery, the results of which he published in 1941 during the Second World War (51, 52). However, Fariñas’ method required surgery.

Yet another decade later, a young resident at Karolinska Institutet in Stockholm by the name of Sven-Ivar Seldinger, M.D. (1921–1998) was working on his doctorate thesis at the Department of Radiology, trying to optimize the insertion of arterial catheters into punctured arterial blood vessels. Seldinger found that – inserting first a guide wire through the needle lumen into the artery, then withdrawing the needle and subsequently inserting a catheter of the same size as the needle via the guide wire – was a fast, safe, and feasible method for arterial catheterization. He published his new method, still known and used today as the Seldinger technique, in 1953 (53). Ironically, his Department Chair deemed this new approach to be insufficient for awarding Seldinger his doctorate (54).

On October 30, 1958, F. Mason Sones, M.D. (1918–1985) at the Cleveland Clinic inadvertently performed the first angiography of a right coronary artery in a 26-year-old patient, establishing coronary angiography as a diagnostic tool and for future developments associated with it (55, 56). He first reported more than 50 cases at the American Heart Association’s Scientific Sessions in 1959 (57). At about the same time, the young surgeon Thomas J. Fogarty, M.D. (born 1934), developed a balloon catheter for removing emboli and thrombi from peripheral arteries (58). Fogarty published the concept for his embolectomy balloon catheter in 1963 (59). The catheter was inserted by surgical arteriotomy, forwarded through and behind the clot, the balloon inflated, and the clot extracted (58). For over a decade, all catheters were hand-made by Fogarty (an example that Grüntzig later would follow) before becoming commercially available in 1969.

## 1964: Charles T. Dotter and Percutaneous Transluminal Angioplasty

Catheter-based percutaneous treatment of occlusive arterial disease had its origins with Charles T. Dotter, M.D. (1920–1985) in the United States (59–63). The first transluminal angioplasty – like the first coronary angiography by F. Mason Sones (55, 56) – was done inadvertently in 1963 (64): while Dotter was performing a routine abdominal aortogram in a patient with renal artery stenosis, he inadvertently re-canalized a stenosed right iliac artery by passing a catheter retrogradely into the occluded artery (64). Following an invitation by Prague radiologist Josef Rösch, M.D. (born 1925), with whom Dotter had been in contact for several years, Dotter reported this accidental recanalization during his lecture “Cardiac catheterization and angiographic techniques of the future” (65, 66) presented on June 10, 1963 at the *Congressus Radiologicus Cechoslovacus* held in Karlovy Vary (Karlsbad). Dotter closed his lecture foreseeing the future use of his method:

«*The angiographic catheter can be more than a tool for passive means for diagnostic observation; used with imagination it can become an important surgical instrument*.» (65).

Rösch later accepted Dotter’s invitation to spend a fellowship at the University of Oregon in Portland, OR, USA, and left Czechoslovakia in March of 1967, only one year before Warsaw Pact troops and tanks occupied his country. Josef Rösch remains active to this day at the Dotter Interventional Institute, established in 1990 at the University of Oregon in the honor of Charles T. Dotter, M.D.

**Figure 2:**
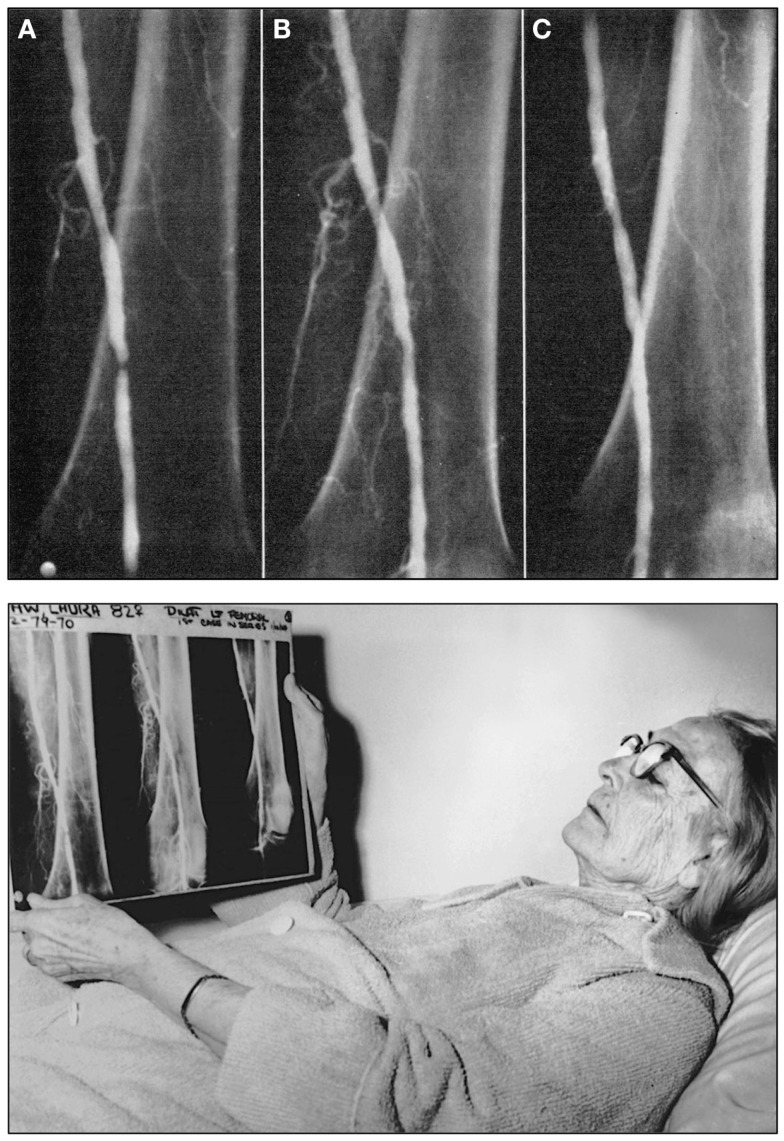
**Top:** Angiograms of the first intentional percutaneous transluminal angioplasty (PTA) performed on January 16, 1964 by Charles T. Dotter, M.D. and Melvin P. Judkins, M.D., in patient Laura Shaw. (A) The initial control angiogram reveals a tight, thread-like stenosis of the left distal superficial femoral artery. (B) Angiogram of the same artery immediately after the angioplasty procedure, which was performed using a 3.2 mm co-axial catheter (C) Follow-up angiogram of the same artery 3 weeks after the PTA procedure demonstrates luminal patency, indicating successful angioplasty. **Bottom:** Charles Dotter’s first angioplasty patient, 83-year-old Laura Shaw in February 1964 three weeks after the first PTA. The patient holds the radiographic films with the same three angiograms shown in the top panel of this figure. Top figure reproduced from Dotter and Judkins (60), with permission of the American Heart Association. Bottom figure reproduced with permission of the Dotter Interventional Institute, Oregon Health and Science University, Portland, OR, USA.

**Figure 3:**
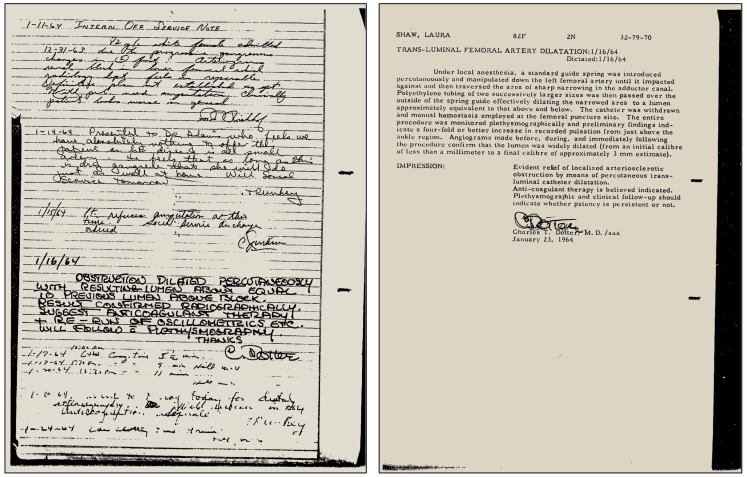
**Left:** Case notes by Charles T. Dotter, M.D., hand-written on January 16, 1964, after the first successful superficial femoral artery PTA was performed in patient Laura Shaw. **Right:** The typed PTA intervention report of January 21, 1964, signed by Dotter. Note: already in 1964 Dotter was using the metric system to indicate lesion size, although in the article reported the same year he was still using inches. Figure reproduced with permission of the Dotter Interventional Institute, Oregon Health and Science University, Portland, OR, USA.

At the time, Melvin P. Judkins, M.D. (1922–1985) had joined Dotter as a radiology fellow at the age of 39 years after having spent more than a decade as a family physician (67). Only shortly thereafter, he would introduce the transfemoral access for cardiac catheterization (67, 68), which would become the standard access route for cardiac catheterization for many decades (67). Six months after his lecture in Karlovy Vary, Dotter and his trainee Judkins performed the first intentional transluminal angioplasty: the patient Dotter had selected was an 83-year-old lady by the name of Laura Shaw (Figure 2) (60, 63, 67, 69), bedridden for months with a cold, painful left leg, and admitted for amputation due to progressive gangrene. A poor run-off arteriogram and the patient’s general condition were thought to contraindicate reconstructive surgery and the patient also refused surgical amputation of her foot (70). After diagnostic angiography on January 6, 1964 had revealed a tight stenosis of the distal superficial artery (60), on January 16, 1964 at 2 p.m., a co-axial catheter system (consisting of a tapered 8 and 12 Fr Teflon^®^ catheter) was introduced to dilate the stenotic area (Figure 2) (60). The angioplasty procedure was successful, the gangrene healed, and the artery remained open until the patient’s death from heart failure 3 years later. Dotter’s hand-written notes on the first percutaneous transluminal angioplasty (PTA) procedure and the typed report are shown in Figure 3.

Dotter published the results of the first patient cases together with Judkins in the same year, and in their article specified further the possible future use of his new method in coronary and renal arteries, at the time applied only to peripheral arteries.

«*Transluminal recanalization appears quite applicable to other arterial systems, particularly those smaller than are usually considered suitable for conventional reconstructive arterial surgery. If its use in femoral disease can be taken as an indication, severe proximal narrowing of the coronary artery will be amenable to a manually guided dilator inserted via aortotomy or via the brachial artery by the Sones technic. Proximal stenosis of the renal, carotid, and vertebral arteries appears suitable for transvascular treatment*.» (60)

Importantly, in his article Dotter already describes in detail the concept of an expandable (dilator/balloon) catheter (“hydraulic dilatation”), which he was developing at the time (60). Dotter mentioned to his patients that he was working on this new catheter, and one patient also reported in his article is quoted that he “anxiously awaits the development of the dilating catheter- probe” (60).

«*In order to improve the technic, a major instrumental design effort is underway. It consists of the development of a device suitable for percutaneous insertion, which is a functional equivalent of the present spring guide but capable of externally controlled concentric expansion over a suitable portion of its length. Expansion from an initial OD* [outer diameter] *of 0.05 to a final OD of 0.2 inch would be desirable. This, it is hoped, will minimize the possibility of inadvertent dislodgement of atheromatous fragments, since the dilator will be positioned in the form of a thin, flexible guide, prior to providing forceful, local expansion as needed*.» (60).

Finally, Dotter presents the concept later to be known as coronary stenting, which at the time he referred to as «endovascular splint», and describes re-endothelialization as “re-intimalization” as the essential regulatory role of vascular endothelial cells in atherosclerosis (8, 9, 71) was unknown at the time.

«*Once a pathway has been created across an occluded segment, repeated dilatation or the temporary use of a silastic endovascular (or, in some cases, paravascular) splint could maintain an adequate false lumen until the natural process of fibrosis and re-intimalization had taken place. We believe re-intimalization is as likely to occur on the walls of a lumen formed by the patient’s own tissues as on the fibers of plastic prosthesis*» (60)

Only one year later, in 1965, Dotter dilated a stenosed iliac artery using an embolectomy balloon developed by Thomas J. Fogarty (72), but concluded that the compliant balloon was not suitable for arterial dilatation and a more rigid one would be needed (64, 72).

Already half a century ago (69), Dotter realized the educational value of film recordings (nowadays video recordings) (73). His 1968 training movie entitled “Transluminal Angioplasty” written, directed, and narrated by Dotter himself on how to perform the angioplasty procedure has been recently released on behalf of the 50th anniversary of its introduction to medicine (69, 73). This movie was to play an important role in the friendship that later developed between Dotter and Grüntzig and will be further discussed below. It is noteworthy that in this movie – like in his 1964 article pioneering angioplasty (60) – Dotter mentions the potential future use of percutaneous transluminal angioplasty in coronary arteries (73). Only 7 years later, Andreas Grüntzig at the University of Zürich would succeed in performing the first experimental coronary balloon angioplasty on September 24, 1975 (74, 75). It was Dotter’s vision and his new approaches that would become an essential element for Andreas Grüntzig’s future work in vascular medicine (76). Ironically, for their different inventive approaches, Dotter and Grüntzig both received a nomination for the 1978 Nobel Prize in Medicine or Physiology (77). The nomination was submitted by William T. Fowley, M.D (1912–1992), a renowned New York vascular physician and clinical expert in the treatment of atherosclerotic vascular disease (78).

## Andreas Grüntzig: Physician and Post-Doctoral Fellow in Heidelberg and London

On April 8, 1964, Grüntzig graduated from medical school at the University of Heidelberg and two years earlier had published his first scientific article which included the data of his D.M. thesis on ventilation effects on dead space changes (79). He wrote his thesis under the supervision of Gotthard Schettler, M.D. (1917–1996), one of Germany’s foremost atherosclerosis researchers at the time (80). After completion of his internship (Medizinalassistentenzeit) in hospitals in Mannheim, Hannover, Bad Harzburg, and Ludwigshafen, by the end of September 1966 Grüntzig returned to his Alma Mater with the goal to pursue a career in public health. Grüntzig’s mentor, former physiologist Hans Schäfer, M.D. (1906–2000) and now Director of the newly founded Institute of Social and Occupational Medicine at the University of Heidelberg, secured him a postdoctoral research fellowship, which also included training in public health and statistics in London and was supported by a scholarship of the Council of Europe (Europarat). Grüntzig later recalled about his research in London:

«*My basic training was in internal medicine. Part of this was taken in London, where I studied the epidemiology of chronic diseases with Geoffrey Rose and Donald Reid. I became particularly interested in atherosclerosis. Later, in 1969, I had the opportunity to meet Dr. Zeitler, a radiologist at a hospital for peripheral vascular disease (Aggertal-Klinik, Engelskirchen, West Germany)*» (81).

In London, Grüntzig worked under Czechoslovakia-born Walter Holland, M.D. (born 1929) (82) at St. Thomas Hospital Medical School, today part of King’s College London, UK, as well as with Geoffrey A. Rose, M.D. (1925–1993) (83), and Donald Reid, M.D. (1914–1977) (84) at the Department of Medical Statistics and Epidemiology of the London School of Hygiene and Tropical Medicine and also completed course in clinical epidemiology (28). Reid’s research was very much focused on atherosclerosis and cardiovascular disease prevention (84), and through Reid Grüntzig learned more about the essentials of scientific excellence, as remembered by Rose (85):

«*Donald Reid’s guiding principles, inculcated in his students, were “to give meticulous care to detail; to know the strength and limitations of one’s data; to form intuitive judgments and then to test them by cold reason; to allow neither laziness nor impatience to erode the determination to get it right; and in all these activities, to be guided by the human values of compassion, integrity, and humility*.”» (85)

In the manuscript of his unfinished autobiography, Grüntzig also indicates that already in the 1960s, he became familiar with atherosclerosis and coronary artery disease from an epidemiological perspective:

«*Three years were spent as a research fellow in the field of epidemiology studying coronary artery disease. I have no doubt that these years dramatically influenced my the ability to incorporate angioplasty into a complex disease process*. […] *I heard Dr. Eberhard Zeitler speaking about peripheral recanalization using Dotter’s method*. […] *I remained very impressed by this new and rather radical approach to the problem of atherosclerosis*» (86)

Grüntzig, who already published several scientific articles during this time dealing mainly with coronary artery disease and its risk factors (79, 87–95), finally decided to leave epidemiology research and pursue a career in clinical medicine. The achievements of his years in Heidelberg and London are summarized in Schäfer’s assessment of Grützig’s work (96):

«*Dr. Andreas Grüntzig was employed as a clinical fellow in the Division of Experimental Occupational Physiology at the Institute of Social and Occupational Medicine of the University of Heidelberg from September 25, 1966 until April 5, 1969. During this time, he was mainly involved in conducting studies for the so-called “Heidelberger Studie”, i.e. organization and clinical investigation of more than 1,000 workers, employees, and clerks of the municipality of Heidelberg. He has processed and analyzed these data with regard to certain aspects and particularly studied the role of alcoholism. In addition, he has tested the reliability of the questionnaires used in the entire study, interpreted clinical data, and performed statistical analyses together with expert statisticians. He has obtained good knowledge of all fields of epidemiological research, and has become acquainted with the most relevant methods of research, both from studying by himself and by attending various courses. He has spent four weeks at the Institute of Demoscopic Research in Allensbach, he worked four weeks with Professor Holland at the Institute for Social Medicine at St. Thomas Hospital in London, completed a course at the London School of Hygiene and Tropical Medicine, which focused on methods of epidemiology and statistics, and finally attended a symposium of the Deutsche Forschungsgemeinschaft which addressed key methodological questions of epidemiology*. […]. *Mister Grüntzig performed all tasks swiftly and with energy, he has gained good knowledge of this area of research, and also performed a literature review to prepare for a study on cerebrovascular atherosclerosis, which, however, could not be conducted due to external factors that had nothing to do with Dr. Grüntzig*.»

[«*Dr. Andreas Grüntzig war vom 25.9.1966 bis 30.4.1969 an der arbeitsphysiologisch-experiementellen Abteilung des Instituts für Sozial- und Arbeitsmedizin der Universität Heidelberg als Assistenzarzt beschäftigt. Er hat in dieser Zeit vorwiegend an der Durchführung der sogenannten “Heidelberger Studie”, d.h. Organisation und Untersuchung von über 1000 Arbeitern, Angestellten und Beamten der Stadtverwaltung Heidelberg mitgewirkt. Er hat die dabei gewonnen Daten in Richtung auf bestimmte Teilprobleme ausgewertet und insbesondere die Frage des Alkoholismus geprüft. Er hat ferner von der gesamten Studie die Zuverlässigkeit der Fragebögen geprüft, Befunde ausgewertet, und zusammen mit Fachstatistikern statistisch verarbeitet. Er hat sich hierbei gute Kenntnisse auf dem gesamten Gebiete epidemiologischer Forschungsmethoden erworben, hat die wesentlichsten Methoden teils aus eigener Anschauung, teils in verschiedenen Kursen kennengelernt. So war er vier Wochen am Institut für Demoskopie in Allensbach, hat vier Wochen bei Professor Holland am Institut für Sozialmedizin des St. Thomas Hospitals in London gearbeitet, hat dann einen Kurs der London School of Hygiene and Tropical Medicine, der sich auf Methoden der Epidemiologie und Statistik erstreckte, absolviert und war schliesslich auf einem Symposium der Deutschen Forschungsgemeinschaft über methodische Grundfragen der Epidomolgie*. […] *Herr Grüntzig hat die ihm übertragenen Aufgaben mit Schwung und Energie angepackt und durchgeführt, hierbei eine gute Kenntnis des Fachgebietes gewonnen, und sich auch für eine Studie über Zereberalsklerose durch Literaturstudium vorbereitet, wobei leider die Durchführung dieser Studie aus äusseren Gründen, die nichts mit Dr. Grüntzig zu tun hatten, nicht zustande kam*.»] (96)

Grüntzig started working as a clinical fellow on May 1, 1969 at the Angiologische Klinik – Max Ratschow Klinik of the Städtische Kliniken Darmstadt, Germany, an hospital specializing in vascular medicine and lead until his death a few years earlier by the founder of German angiology, Max Ratschow, M.D. (1904–1963) (97, 98). At the Angiologische Klinik – Max Ratschow Klinik (today Max Ratschow Klinik für Angiologie – Medizinische Klinik IV as part of the Klinikum Darmstadt), Grüntzig became familiar with the clinical picture of peripheral artery disease. It was at the Klinik Max Ratschow where Grüntzig and Ernst Schneider, M.D. first met (99). One of Grüntzig’s patients asked him whether it was possible – instead of using drug treatment or undergoing complex coronary bypass operations – to just “clean” his obstructed arteries, like a plumber cleans tubes using wire brushes. Grüntzig found this idea “*fascinating*” (37, 96). According to Grüntzig’s own words (96), this was the moment when he began to develop his first theories about therapeutic vascular interventions. Unlike previously reported (100, 101), Grüntzig performed his first angiographies of peripheral arteries already in Darmstadt in 1969 (and not in Zürich in 1971); in his training certificate issued on October 10, 1969, by the head of the angiology department, H. M. Hasse, M.D. (96), Hasse attested Grüntzig:

«*In addition, he learned angiographic techniques and with great skill successfully performed angiographies of peripheral arteries, aortographies, and phlebograpies*. […] *In his work he demonstrated agility and a quick mind. His good predisposition and many interests qualify him for the medical profession and for academic medicine in particular*»

[*“Darüber hinaus erlernte er die angiographische Technik und führte mit Geschick zahlreiche Extremitäten-Angiographien, Aortographien und Phlebographien erfolgreich durch*. […] *In seinen Arbeiten bewies er Wendigkeit und schnelle Auffassung. Seine guten Anlagen und seine vielseitgen Interessen befähigen zum Arztberuf und zur wissenschaftlichen Laufbahn im besonderen Masse.”*] (96)

During this time, Grüntzig happened to attend an afternoon meeting of lectures given by the «Frankfurter Angiologischer Kreis» [«Frankfurt Vascular Medicine Circle»], organized by Dieter Gross, M.D., Wolfgang Rotter, M.D., and Klaus Breddin, M.D., some of Germany’s leading vascular physicians at the time (102). During that afternoon, Eberhard Zeitler, M.D. (1930–2011), of the Aggertal-Klinik in Engelskirchen, presented his experience and clinical success using the new Dotter angioplasty procedure (103). On his way from the meeting back to Darmstadt, Grüntzig asked his department head H. M. Hasse, M.D. for permission to learn more about the Dotter procedure that Zeitler had established at the Aggertal-Klinik (102–104). Grüntzig later told Zeitler that Hasse made it very clear to him that there wasn’t a chance:

«*I will never allow this kind of technique to be practiced at my hospital*»

[*“So eine Technik kommt mir nicht ins Haus”*] (102).

Also in the manuscript of his unfinished autobiography, Grüntzig recalls the Frankfurt meeting and Hasse’s response to his request:

«*It was at this time that my chief invited me to attend an afternoon meeting in Frankfurt. For the first time, I heard Dr. Eberhard Zeitler speaking about peripheral recanalization using Dotter’s method. I recall that my chief was very upset that someone would try to attack diseased arteries by forcing catheters into areas of narrowing. He made it clear that he never wanted such a treatment to take place in his hospital*.» (86)

## Andreas Grüntzig: Developing Vascular Medicine at the University of Zürich

“I have dedicated my life to vascular disease”

Andreas Grüntzig, M.D. (1969)

**Figure 4:**
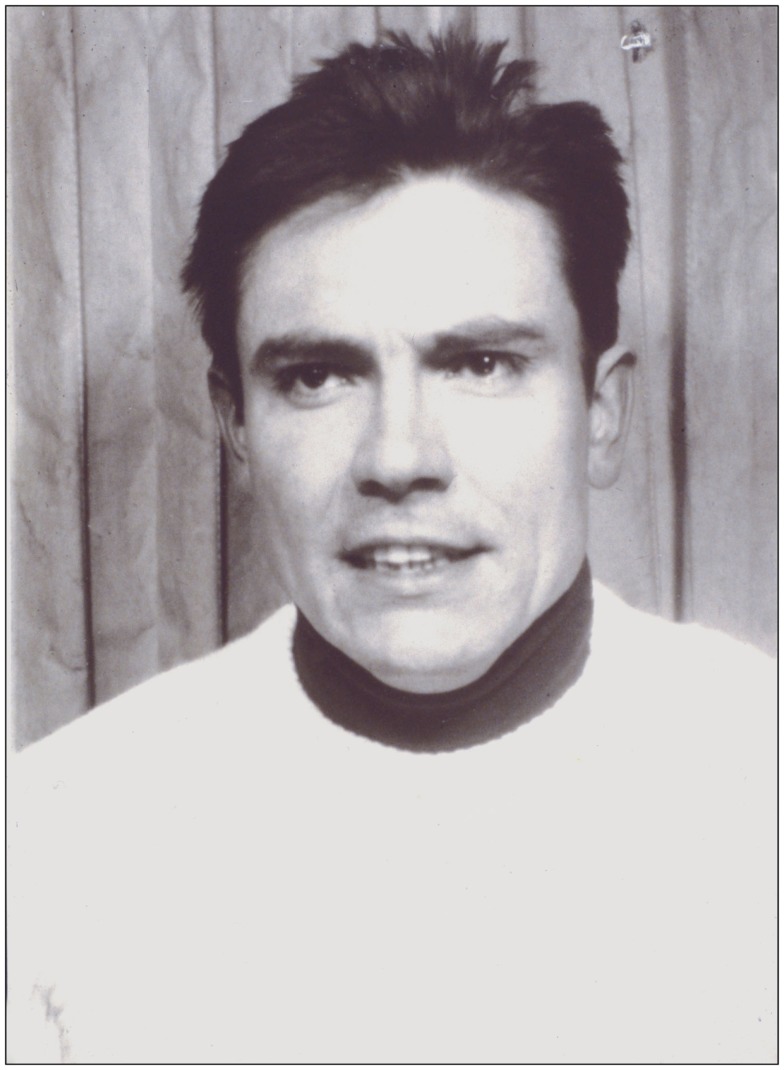
**Photograph of Andreas Grüntzig taken in 1969 at the time when he was recruited by Robert Hegglin, M.D., to join the Medical Policlinic at the Kantonspital of the University of Zürich**. Photograph reproduced with permission of Ernst Schneider, M.D., Zürich.

During his medical studies in Heidelberg, Grüntzig had come across «*Differentialdiagnose Innerer Krankheiten*», a widely popular internal medicine textbook written by Robert Hegglin, M.D. (1907–1969). Hegglin, Editor-in-Chief of the journal *Cardiology* from 1962–1969, was Director of the Medical Policlinic at the Kantonspital of the University of Zürich and published his monograph «*Differentialdiagnose Innerer Krankheiten*» between 1952 and 1969 in 11 editions, many of which were translated into foreign languages (105). His successor, Walter Siegenthaler, M.D. (1923–2010), would later continue its publication using Hegglin’s original title, albeit publishing Hegglin’s book under his own name (106). With his book, Hegglin’s followed an approach introduced by William Osler, M.D. (1849–1919) who 60 years earlier had also published an internal medicine monograph for medical students and practicing physicians (107). Hegglin had trained under Wilhelm Löffler, M.D. (1887–1972), who founded the journal *Cardiology* in 1937 and was its Editor-in-Chief until 1962. Hegglin dedicated his book to Löffler acknowledging him as his teacher (105). Hegglin was known as an outstanding clinician and educator. He is still known today for the discovery of the May–Hegglin anomaly and the Fanconi–Hegglin syndrome (108, 109). Grüntzig became fascinated by Hegglin after reading and studying with his book (105); he was determined to pursue his clinical training with Hegglin and applied for a position as a clinical fellow (Figure 4). When Hegglin invited Grüntzig for a personal interview in 1969, he asked him about his future plans. Grüntzig’s response was clear and determined: “*I have dedicated my life to vascular disease*” (99–101). Hegglin hired Grüntzig who began working as a clinical fellow in the same year. As there was no open position in Hegglin’s Medical Policlinic, Grüntzig first worked in the recently founded Division of Angiology lead by Alfred Bollinger, M.D. (born 1932). Hegglin died unexpectedly in the same year of a ruptured aortic aneurysm.

Bollinger soon recognized the exceptional professional qualities of Grüntzig, who worked into the night to help Bollinger with his research papers (99). Bollinger remembered:

«*If I asked Andreas to write an article, I’d have it in ten days. If I asked for revisions I’d have them in two. He was quick and absolutely decisive”*. […] *He was always very rigorous with these kinds of things in seeking reliability and reproducibility. He was very exact and precise and concerned to make things statistically relevant. I have never worked with anyone in medicine who had the kind of inner fire that he had. It was unbelievable*.» (99).

## Translational Vascular Medicine in the 1970s: The Grüntzig Balloon Catheter

*“… and refinements would probably be suggested by others as well as us. What’s needed is a working beginning*.

*YOU OF ALL PEOPLE MUST KNOW HOW WIDE THE GAP BETWEEN THE IDEA + THE GADGET. IT’S THE LATTER WHICH PAYS OFF*.

I actually made heat-shrink, preformed sleeve balloons for catheters once but I wasn’t professional or self confident.”

Charles T. Dotter, M.D.

In the 1960s, Charles T. Dotter, M.D. – as reflected by his above quote from a communication with Bill Cook (69) – had also experimented unsuccessfully with sleeve balloons. In fact, when Fogarty was an intern at the University of Oregon in the 1960s, Dotter asked Forgarty to make some balloons (110) and even once used an embolectomy balloon to dilate an iliac artery (64, 72). However, Dotter could not develop a sufficiently rigid balloon catheter. To improve angioplasty of larger arteries, Dotter in 1966 had constructed a “reinforced balloon-dilating catheter” in which a woven fiberglass sheath surrounded a simple balloon catheter (70). Grüntzig knew also of studies done with so-called caged balloon catheters («Korsett-Ballonkatheter») introduced in 1973 by Werner Porstmann, M.D. (1921–1982) in Berlin. This catheter consisted of an 8-Fr outer Teflon^®^ catheter with four longitudinal slits. A latex balloon catheter inflated inside the slits permitted dilation up to 9 mm, sufficient to treat iliac artery lesions, yet it did not yield the expected results (111). Dotter improved Porstmann’s device and used the “caged balloon-dilating catheter” for successful treatment of iliac artery obstructions in 48 patients (112). Early failure with thrombosis, however, limited the use of this dilating catheter and neither of these balloon catheters ever found its way into wider practice (70). Despite unsuccessful attempts of his colleagues, Grüntzig remained optimistic and was convinced that the idea to apply defined pressure from the luminal side using an inflatable balloon to the stenosed vascular wall would be the way to make his idea work.

In 1971, after Grüntzig had moved to the Department of Radiology of the University of Zürich working under Josef Wellauer, M.D. (1919–1997), he was again able to perform arterial angiographies, which he had started 2 years earlier in Damstadt at the Max Ratschow Klinik. Grüntzig also visited Zeitler at the Aggertal-Klinik in Germany, where he familiarized himself with the Dotter technique (28, 76). Grüntzig remembered:

«*I not only observed the procedure itself during this time but also saw the patients before and after treatment and when they left the hospital. I was very impressed with the improvement in peripheral ankle pressure as measured by ultrasound and by the fact that the patient was able to walk without any claudication after successful catheter treatment*.» (86)

Grüntzig invited Zeitler back to Zürich to introduce the “dottering” of occluded arteries (dilating arteries using the Dotter-catheter technique, or “*Dottern*,” as the German physicians called it). In the same year, on December 15, 1971, Grüntzig began performing angioplasties using Dotter catheters in Zürich, the first of about 50 cases that would follow. Grüntzig published the results of the first Dotter angioplasty studies in Zürich in 1973 (113).

## 1974: The First Balloon Angioplasty at the University of Zürich

According to Grüntzig (22), the idea for the balloon catheter was born during the same time when he began to use Dotter catheters in Zürich, i.e., in late 1971. Within another 2 years after his arrival in Zürich and without any support from the federal funding agencies, working at home in the evenings and on weekends in addition to his full-time clinical duties, Grüntzig had succeeded in having functional, hand-made balloon catheters at his disposal (22).

In October 1973, Grüntzig moved to the Division of Cardiology of the Kantonspital’s Medical Policlinic, at the time headed by Wilhelm Rutishauser, M.D., but continued doing angioplasty procedures in patients at the University’s Department of Radiology. Four months later – on February 12, 1974 – Grüntzig for the first time successfully applied his new single-lumen dilating catheter with a 4 mm balloon in his patient Fritz Ott, a 67-year-old gentleman who had been admitted because of incapacitating claudication caused by a severe femoral artery stenosis (99, 101). Bollinger remembered how Grüntzig obtained informed consent from his patient:

«*In his kind and caring way, Andreas explained the therapeutic principle to the patient during a full hour and obtained his informed consent, long before ethical committees existed*.» (99).

**Figure 5:**
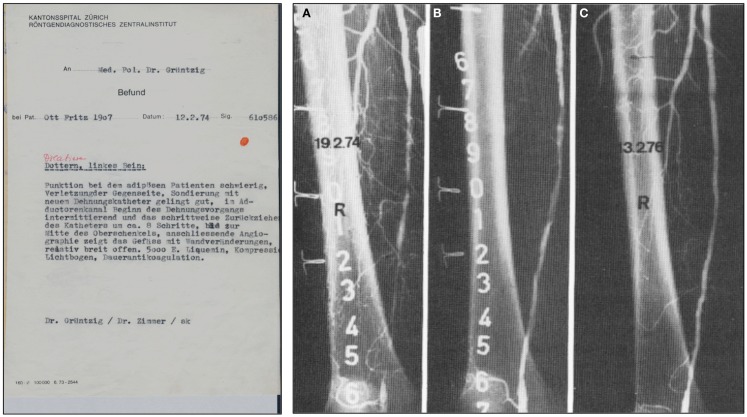
**Left:** A copy of Grüntzig’s report written in German of the first balloon angioplasty performed on February 12, 1974, utilizing his hand-made, single-lumen balloon catheter. In this report, Grüntzig describes “Dottern, linkes Bein” [Dottering, left leg], and summarizes the procedure. Grüntzig refers to a “neuer Dehnungskatether” [new dilatation catheter], which is the only hint that a balloon angioplasty was performed. To specifically indicate this, Maria Schlumpf added “Dilation” in red ink above the description of the procedure right after the typed report had become available (39). **Right:** Femoral artery dilatation by Grüntzig performed on February 19, 1974 using a single-lumen balloon catheter. (A) Severe atherosclerotic disease resulting in several stenoses of the right superficial femoral artery, note severe wall irregularities. (B) The same artery after balloon dilation: Grüntzig described the PTA procedure as follows: *“We dilated the entire artery down to the knee. The step-by-step dilatation of the superficial femoral artery resulted in a hemodynamically sufficient lumen, but severe wall irregularities are visible after dilation. After 2 years the vessel lumen is completely clear. Since the intervention, the patient has been symptom free.”* (81) (C) In the dilated artery, patency was confirmed two years later. Photographs reproduced with permission of Maria Schlumpf, Zürich.

Grüntzig’s typed report of this first successful balloon angioplasty and the angiographs of another early femoral artery balloon dilatation performed in a 74-year-old patient a week later are shown in Figure 5. Though Grüntzig refers to his balloon new device as “new dilatation catheter” (neuer Dilatationskatheter), he described this first balloon PTA procedure simply as “Dottern.” To indicate that this was the first ever *balloon* dilatation, Grüntzig’s assistant Maria Schlumpf in red ink at the time added to the report the word “Dilatation” (Figure 5) to indicate that the procedure was different from the regular Dotter-catheter procedures that Grüntzig was also routinely doing at the time. Like Dotter’s first patient Laura Shaw 10 years earlier (Figure 2), Grüntzig’s first patient Fritz Ott was soon able to walk again free of pain (99–101).

**Figure 6:**
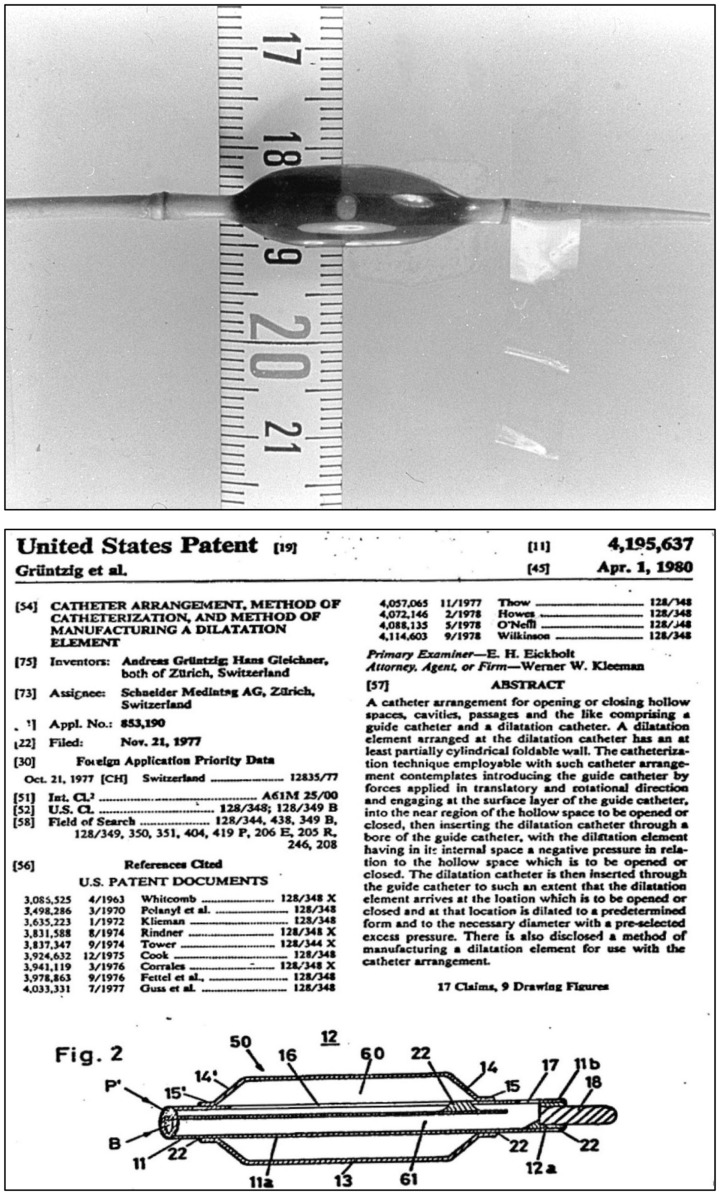
**Top**: Photograph of the inflated, original balloon-dilating catheter hand-made by Andreas Grüntzig, which he used for the first iliac artery balloon angioplasty on March 6, 1974. For size comparison, a centimeter tape measure is shown. Angiograms obtained during this PTA procedure are available online in another article of the Journal (26). **Bottom**: Part of the first page of the U.S. patent application by Andreas Grüntzig and Hans Gleichner, also showing a cross-section drawing of the balloon catheter, which resembles the original device shown in the upper panel of this figure. Top photograph reproduced with permission of Ernst Schneider, M.D., Zürich.

Less than a month after the first femoral balloon angioplasty, Grüntzig performed the first balloon angioplasty of an iliac artery stenosis on March 6, 1974 using a shorter but wider balloon measuring 8 mm in diameter (the tip of the original catheter used is shown in Figure 6). Pre- and post-interventional angiograms of this first iliac artery dilatation were included in the initial report by Grüntzig and fellow German Heinrich Hopff, Ph.D. (1896–1977) (22). The angiograms of Grüntzig’s first iliac artery balloon angioplasties were re-published in our recent report in the Journal (26). Grüntzig described the first iliac artery balloon dilation as follows:

«*A 73-year-old male patient had hyperlipidemia, hypertension, diabetes, and myocardial infractions with intermittent claudication of 500 m. He was considered a candidate for dilation. We started the series of iliac dilations and used our new iliac dilation catheter for the first time in March 1974, in this patient. He became symptom free until the progression of disease in the right femoral artery. The figure* [lower panel (114)] *shows the compression of the atheroma against the medial site of the common iliac artery. The disease did not recur in the dilated segment until 5 years later*» (81).

Hopff was an expert in the chemistry of polyvinyl chloride (PVC) and an emeritus professor of organic chemistry at the Federal Institute of Technology (ETH) in Zürich. Based on the results of his research (114, 115), he suggested to Grüntzig to use PVC as material of high rigidity that might be suitable for the use as a dilating balloon. In the manuscript of his unfinished autobiography, Grüntzig describes meeting Hopff and his search for the ideal balloon material:

«*I spent the next two years contacting manufacturing plants in an attempt to solve this problem. Especially fruitful was the cooperation of a factory which produced shoelaces which provided me with silk meshes which I planned to wrap around the balloon, thus limiting its outer diameter. I then needed a very thin balloon to insert within the mesh. It was at that time that I met a retired chemist, Dr. Hopff, a professor emeritus of chemistry* […] *. He introduced me to polyvinyl chloride compounds. I started to experiment with this material and studied his book on organic chemistry. I acquired some small thin polyvinyl chloride material used as insulation for electrical wires. Following the descriptions in his book* [(115)] *, I heated a localized segment of the tubing and applied compressed air pressure resulting in a localized aneurysm of the tubing. I used a second outer tubing measuring 4 mm in diameter to confine the diameter of the segment. After hundreds of experiments, most of which were performed in my own kitchen, I was able to form a sausage-shaped distensible segment which I tried to reinforce with the silk mesh. When I mounted the material on a normal catheter tubing and applied pressure to distend the aneurysmal segment, I suddenly realized that the strength of this material was so great that the silk mesh was not necessary. This was a great breakthrough and enabled me to reduce the size of the catheter*.» (86)

**Figure 7:**
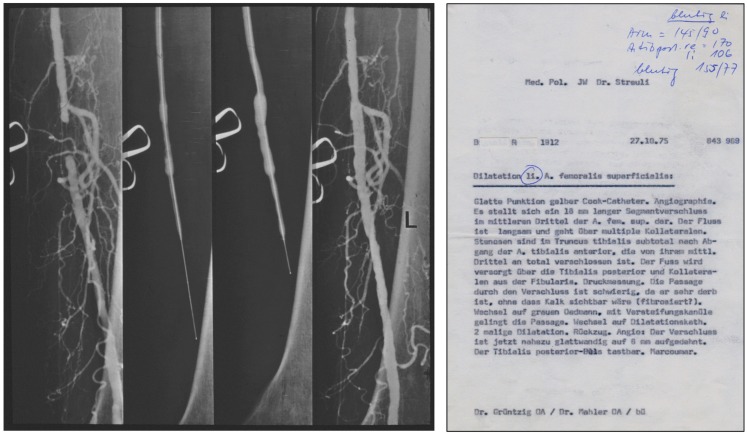
**Left:** Pre-, inter-, and post-interventional angiograms of balloon PTA of an occluded left superficial femoral artery in a 63-year-old male patient, performed by Grüntzig and Mahler on October 27, 1975. **Right:** A copy of the report summarizing the PTA procedure of October 27, 1975. Femoral artery angiography demonstrates an occluded segment measuring 18 mm in length. Passage of the occlusion is difficult, but possible after changing to a special catheter (gray Ödman catheter). The operator changes to the balloon catheter [“Wechsel auf Dilatationskatheter”]; subsequently, the occluded segment is dilated twice and blood flow successfully restored. Photographs reproduced with permission of Maria Schlumpf, Zürich.

**Figure 8:**
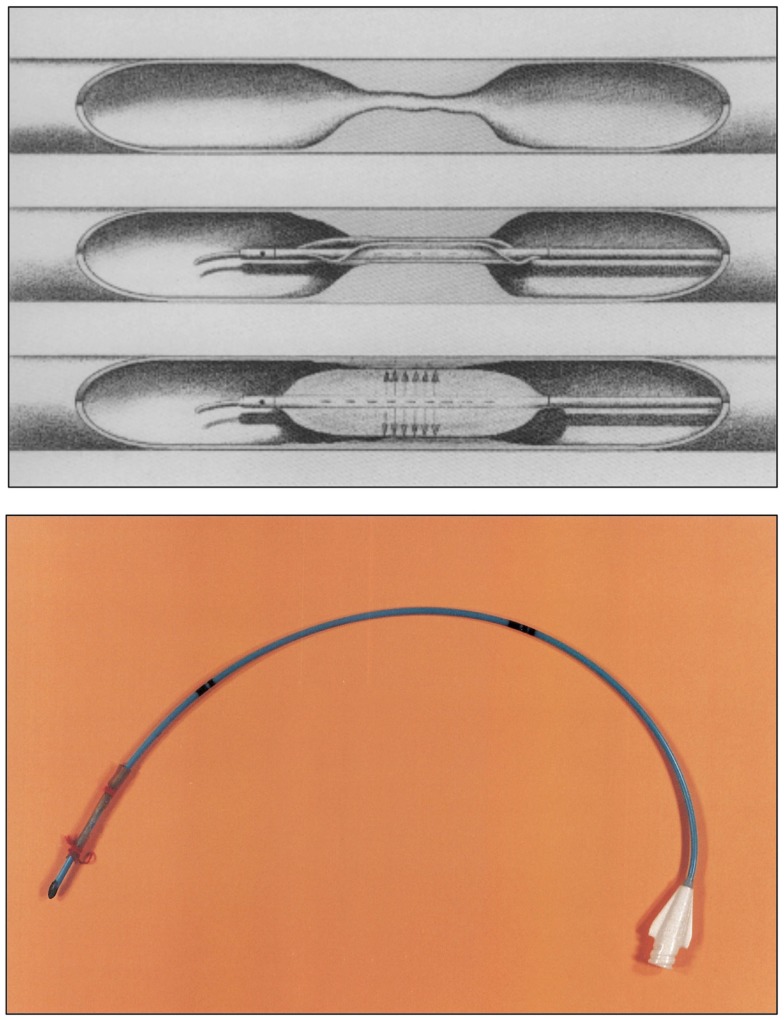
**Top:** the Grüntzig balloon catheter principle. The double-lumen dilatation catheter (120) now contains a main lumen and an additional lumen. The main lumen allows insertion of the guide wire, pressure measurements, and injection of contrast dye. The balloon segment at the catheter tip can be positioned in the stenosed or occluded vascular segment and is filled with liquid via the additional lumen. By applying an equally distributed and constant pressure between 4 and 6 atmospheres, the atherosclerotic plaque occluding the artery is pressed against the vessel wall for 10–30 s. The maximum diameter of the inflated balloon is 4 mm for femoral arteries and 8 mm for iliac arteries. Once the balloon is deflated, the newly formed vascular lumen opens, and blood flow is restored. In its deflated form, the balloon will adhere to the catheter like an umbrella and can be pulled back, and the procedure is completed. Figure legend and drawing according to a sketch by Andreas Grüntzig (36). **Bottom:** the very first balloon catheter constructed by Grüntzig in 1971, long before balloon catheters were fully functional for the use in patients. The tip of this single-lumen catheter was sealed to allow inflation and extension of the balloon, which was glued and fixed on both sides with a thread onto the catheter tube. Balloons were fixed with surgical sutures to all hand-made catheters until mid-1975 when Cook and later the Schneider company began manufacturing Grüntzig balloon catheters. Photographs reproduced with permission of Maria Schlumpf, Zürich.

Grüntzig and Hopff published the principle of the new balloon catheter and reported the clinical findings from a series of the first 15 patients successfully treated with balloon angioplasty (22, 26). We have recently discussed how the international recognition of this method was delayed because the article was published in German only (26). Grüntzig initially coined his technique “percutaneous transluminal dilatation” (116). In his habilitation thesis, submitted to the University of Zürich in 1977 and qualifying him for faculty rank, Grüntzig referred to his method as “percutaneous transluminal recanalization” (116), a term previously introduced by Dotter (60) and also used by others (117). For the cover of his habilitation thesis, which he published as a book (116), Grüntzig selected pre- and post-interventional angiograms of a balloon catheter-treated femoral artery occlusion, which he successfully re-opened on October 27, 1975, these angiograms are shown in Figure 7. The Grüntzig balloon angioplasty procedure became known as PTA or as “percutaneous transluminal coronary angioplasty” (PTCA) in cardiology (118), with reference to the terminology originally introduced by Dotter (60, 64, 73). Grüntzig filed a patent application on the balloon catheter concept with the U.S. Patent Office on November 21, 1977 (Figure 6) and simultaneously with patent offices in Switzerland, Germany, France, England, and Japan (26), securing the rights to his intellectual property of the balloon catheter principle (Figure 8). The bottom panel of Figure 8 shows the first balloon catheter ever made by Grüntzig at a stage when it was not yet intended for patient use.

## Back into the Future: Personalized Medicine in the 1970s

**Figure 9:**
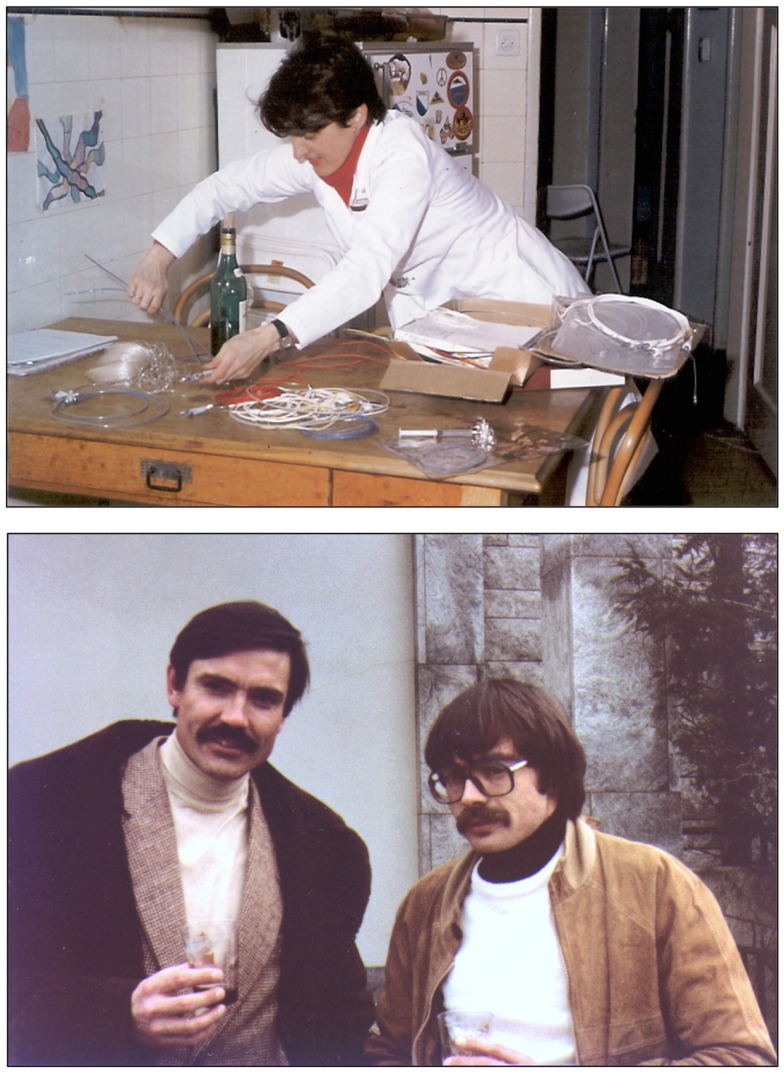
**Top:** Maria Schlumpf at the Grüntzig kitchen table sorting out materials used for building the hand-made balloon catheters. Catheters were built by both of them with the help of their spouses Michaela Grüntzig and Walter Schlumpf. Photograph ca. 1975. **Bottom:** Andreas Grüntzig and Pierre Levis in Atlanta, GA, USA, during the 1981 angioplasty course held at Emory University. Photographs reproduced with permission of Maria Schlumpf, Zürich.

For the next two and a half years, each balloon catheter would be custom-made for each individual patient on the Grüntzig kitchen table (Figure 9) (36). Prior to the PTA procedure, Grüntzig would obtain a native angiogram, take it home, measure the arterial lumen and length and width of the stenosis from the angiogram, calculate the optimal diameter and length of the balloon needed, and build a balloon to fit that particular stenosis (39); he would accomplish this together with his assistant Maria Schlumpf and the help of their spouses, Michaela Grüntzig and Walter Schlumpf. Thus, there was «*personalized medicine*» already in the early 1970s. The first single-lumen balloon catheters (used until January 1975) were red “Ödmans” [catheters], in which little holes were cut using razor blades in those portions of the catheter where the balloon was to be inflated. The PVC balloon tube was glued to the catheter and both ends were fixed with a fine thread; the knots of the threads tying the balloon tube to the catheter were always done by Grüntzig himself, who would justify this with his surgical skills being the only physician on the team (39). All balloon catheters were used only once, and until the second half of 1976, when the Schneider company was founded and began to manufacture Grüntzig’s balloon catheters, every catheter used for balloon angioplasty procedures in Zürich was a hand-made, custom-built device manufactured in the evenings and on weekends (36) (Figure 9).

## 1977: The First Coronary Balloon Angioplasty

**Figure 10:**
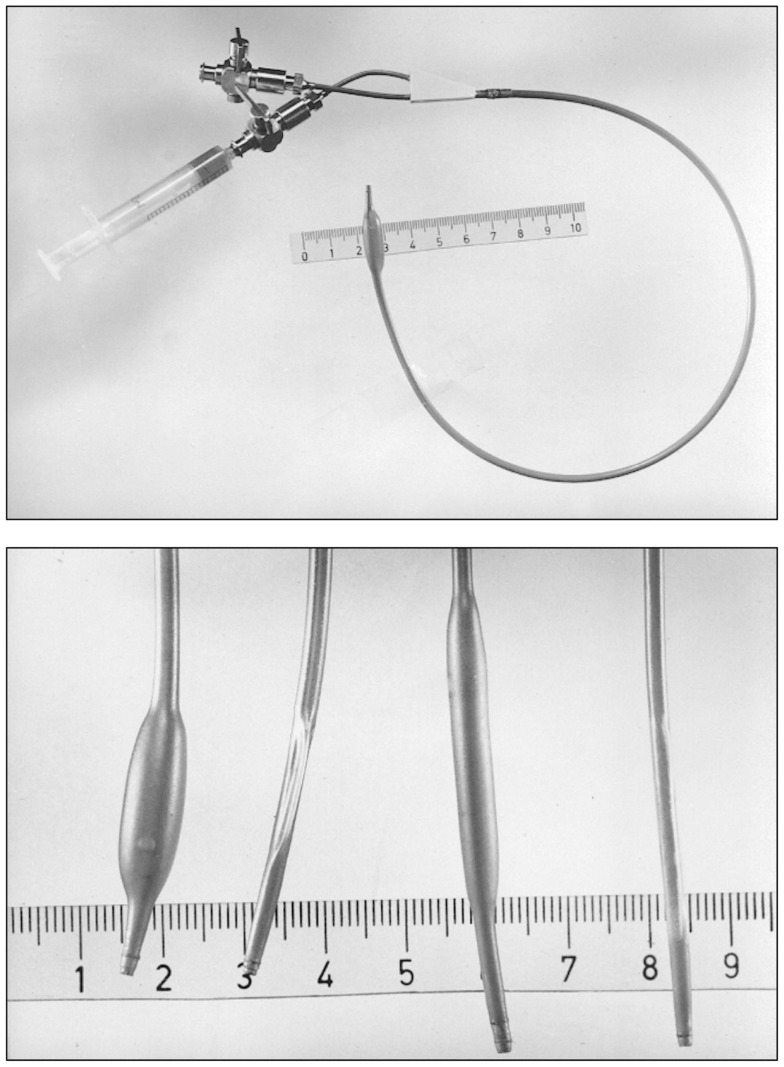
**Top:** A double-lumen catheter for iliac artery balloon angioplasty, connected to a three port stop-cock, with one port connected to a syringe containing liquid to inflate the distensible balloon portion of the catheter. Tape measure indicates centimeters. **Bottom:** Hand-made double-lumen balloon catheters assembled by Grüntzig and his associates on his kitchen table shown in Figure 9. Shown on the left is an inflated iliac artery balloon catheter (with a similar, deflated catheter to its right) and an inflated femoral artery catheter (with a similar, deflated catheter to its right). Note that the balloons are tied to the catheters with fine surgical threads and that catheter tips have been conically tapered, which was done by hand using sandpaper. Inflated balloon diameter of femoral catheters is 4 and 8 mm in iliac artery catheters. Tape measure indicates centimeters. Photographs reproduced with permission of Maria Schlumpf, Zürich.

**Figure 11:**
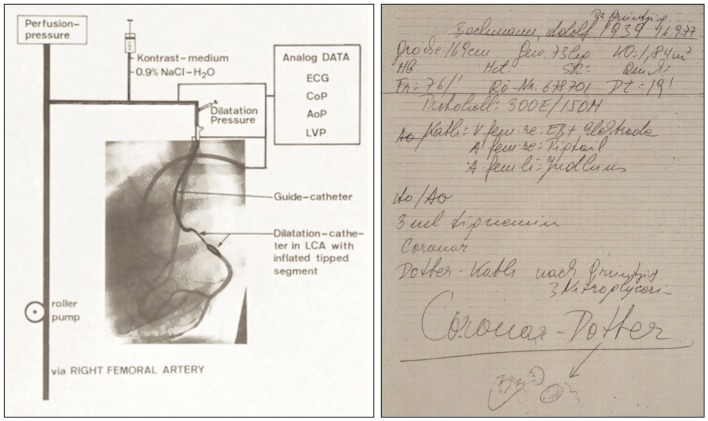
**Left:** Schematic representation of the coronary double-lumen balloon dilatation catheter used for PTCA procedures of stenosed canine coronary arteries, which Grüntzig published in 1976 (74, 121, 124). The preformed guiding catheter is sealed by an adaptor against the dilatation catheter localized inside. The main channel of the double-lumen catheter is connected to a three port stop-cock, which can be used to either measure coronary pressure, to inject contrast dye, or to perfuse the coronary artery with arterialized blood. Connected to the neighboring channel of the dilatation catheter is a syringe, which is used to inflate the distensible segment of the dilatation catheter. Pressure is applied by filling the distensible segment, which exhibits only minimal compliance. To demonstrate perfusion with arterialized blood, contrast dye was injected via the main channel, resulting in visible filling of the circumflex branch of the left coronary artery. Additional catheters positioned in the left ventricle and the aorta are also connected to the central recording unit. AoP, aortic pressure; ECG, electrocardiogram; CoP, distal coronary artery pressure; LCA, left coronary artery; LVP, left ventricular pressure. **Right:** Grüntzig’s notes taken by the nurse assisting him with the first coronary balloon angioplasty performed a conscious, awake patient on September 16, 1977, in Zürich, Switzerland. Four months earlier, Grüntzig had already performed several coronary balloon angioplasties after coronary arteriotomy during open heart surgery together with Richard K. Myler, M.D. and Elias Hanna, M.D. at St. Mary’s Medical Center in San Francisco. At the bottom of the note, there is a small drawing of the aortic root with the coronary arteries, indicating with an arrow the portion of the proximal LAD where Grüntzig performed what he refers to as the first “*Coronar-Dotter*,” paying tribute to the inventor of endovascular therapy. On this note, the catheter used for the procedure is referred to as “*Coronar-Dotter-Kath. nach Grüntzig*” [“*Coronar-Dotter catheter according to Grüntzig*”]. Left photograph reproduced with permission of Maria Schlumpf, Zürich, right photograph reproduced with permission of Ernst Schneider, M.D., Zürich.

Soon, Grüntzig would develop a double-lumen catheter allowing greater versatility and better handling than single-lumen devices, and he commissioned engineer Helmuth Schmid to add a second groove into the red Ödman catheters (39). The first femoral angioplasty using a double-lumen balloon catheter was performed on January 23, 1975 (119), and Grüntzig published his experience with this new device in 1976 (120) (Figure 10); ironically, the article abstract does not mention the word “balloon” but only describes the new double-lumen balloon catheter as “a modification of Dotter’s transluminal recanalization of stenoses and occlusions” (120). Grüntzig was now able to apply equal, *constant and defined* pressure to the vascular wall (Figure 8) as he had planned in 1971. While Grüntzig continued to treat patients with peripheral vascular disease using new, double-lumen balloon catheters (Figure 10), he also started to explore other vascular territories. In 1974, Grüntzig began developing and experimenting with catheters of reduced diameter that would allow their use in coronary arteries (Figure 11).

Grüntzig was aware that others, including Dotter – who already mentioned the coronary circulation as a potential target for angioplasty in his 1964 article (60), had thought of a broader application of angioplasty including the coronary arteries, and specifically acknowledged Dotter’s pioneering role in the field (29). Grüntzig remembered:

«*My background in internal medicine was most helpful in helping me follow the patient’s progress clinically. At that time it was my intention to become a cardiologist and I began to consider application of the technique to the heart. I designed a prospective follow-up study of the patients treated with the Dotter technique with regular examinations every three months after the procedure in order to begin assessing the long-term patency of these patients. The early results were favorable but from the very beginning I realized the problems and limitations of Dotter’s technique. Everyone involved in the method at that time, including Charles Dotter, realized that any application of the dilatation procedure to other areas of the body would require technical changes*.» (86)

In Zürich, the cardiac surgeon Åke Senning (36, 38, 39) – unlike Siegenthaler, Krayenbühl, or the surgeon Hans-Ulrich Buff (38) – fully supported and actively helped Grüntzig to proceed with his plans. In fact, when Grüntzig approached him asking whether he could count on his support, Senning responded:

«*Mr. Grüntzig, you will be taking away my patients, but get started right away!*»

[«*Herr Grüntzig, Sie werden mir die Patienten wegnehmen, aber legen sie los!*»] (38)

With Senning’s support, Grüntzig began testing smaller balloon catheters for coronary arteries (Figure 11) in large animals (36, 121, 122) and human cadavers, with the help of his Swiss assistant, Maria Schlumpf, and the Croation cardiac surgeon Marco Turina, M.D. (born 1937) (122), and others. Turina, who at the time Senning had commissioned to perform the cardiac surgery in the experimental studies (74), remembers Grüntzig:

«*He had the ‘sacred fire’, as the French call it. It was what he thought about constantly. I have never seen somebody so centered on a single idea like Andreas was. Never in my life. Everyone was telling him his idea would never work, and had been tried before, and that he was going to fail, that there were pitfalls at every turn. But the idea was consuming him all the time*.» (99).

Grüntzig’s younger daughter Sonja, who recalls having her meals at the same kitchen table that the catheters were made on, joked about herself being a “twin” of the Grüntzig balloon catheter, as her father was completely absorbed with making his idea work (123).

Grüntzig began to evaluate new, more refined and thinner coronary balloon catheters in canine coronary arteries (74, 121, 122) in which the surgeons had experimentally induced coronary stenoses (Figure 11). The first balloon dilatation of a coronary artery was performed in Zürich on September 24, 1975 (119). The data from these experiments – demonstrating the feasibility and efficacy of this approach – were presented at the Spring meeting of the Deutsche Gesellschaft für Kreislaufforschung (today Deutsche Gesellschaft für Kardiologie, DGK) in Bad Nauheim, Germany, in April 1976 (74), as well as at the American Heart Association’s Scientific Sessions in Miami, FL, USA, November 15–18, 1976 (124) (Figure 11). Given the success of coronary balloon angioplasty in his experimental studies, Grüntzig was determined to test its feasibility in coronary artery disease patients (99, 116). Only now, having successfully completed translational research that had already resulted in a new, non-surgical treatment of occluded peripheral arteries and established coronary angioplasty in experimental studies (74, 121, 122), Grüntzig applied for research funding from the Swiss National Science Foundation (SNSF). Within the next 4 years, he was awarded three SNSF research grants as Principal Investigator (125–127).

As the first successful renal angioplasty had been performed by Zeitler at the Aggertal-Klinik in 1970, using co-axial Dotter catheters (29, 128), Grüntzig also began to explore this vascular territory. In Zürich, on February 2, 1977, Grüntzig for the first time inserted his balloon catheter into a stenosed human renal artery, and into a human coronary artery on February 16, 1977; in both cases, Grüntzig did not perform angioplasty (39). In December 1977, Grüntzig at the University of Zürich (29) and Mahler at the University of Bern (129) performed the first renal artery PTA using Grüntzig balloon catheters.

On March 22, 1977, Grüntzig was called to explore the feasibility of his new coronary balloon catheter for percutaneous transluminal coronary angioplasty (PTCA) a patient with severe multi-vessel coronary artery disease, including a left main stenosis, which the surgeons deemed inoperable. He failed to gain femoral access – an approach pioneered by Melvin P. Judkins, M.D. in 1967 at the University of Oregon (67, 68) – trying to reach the coronary artery ostium using a brachial access. Grüntzig recalled the first PTCA attempt in humans, already here stressing the issue of patient safety:

«*The first patient on whom we attempted dilatation was a case in which coronary vascular surgery was denied. The patient with unstable angina, multivessel disease, mainstem stenosis, referred to the coronary care unit, was presented to me and everyone assured me that attempted dilatation with success would be the proof for the efficacy of this method. I agreed, eager to enter the area of competition with the surgeons. Unfortunately the patient was so diseased that every attempt to puncture the groin arteries failed because of total closures; only the left brachial artery had positive flow to allow the passage of the catheter to the aorta. With this entrance, the catheters were unable to guide the coronary dilatation catheter to the orifice of the left main so that we had to abandon the procedure. The patient died several days after the procedure of a final myocardial infarction. The case taught me that if you start a method, you should start with an ideal case and not with end stage disease and this has been the truth for so many other colleagues being in a similar position later in time*.» (86, 130)

As the application of the new technique to human coronary arteries would be challenging and in order to allow development of the PTCA procedure in a safe manner, Grüntzig insisted PTCA to be performed during bypass surgery so that patients would not be jeopardized (131). In the manuscript of his unfinished and unpublished autobiography, Grüntzig wrote in 1985:

«*The next step was to demonstrate the technique in intra-operative dilation. Cardiac surgeons in Zurich resisted the application of intra-operative dilatation because they feared that retrograde dilatation of the underlying stenosis in the native coronary artery would create a competition in blood-flow to the vein graft therefore resulting in closure of the graft. They contended that the long-term patency of the dilated native coronary stenosis was unknown and therefore presented a risk to the patient*.» (86)

Thus, Grüntzig began looking elsewhere. Within a short time, Richard K. Myler, M.D. (1936–2013) and Grüntzig convinced Elias Hanna, M.D. (born 1936) (132), a cardiac surgeon at St. Mary’s Hospital in San Francisco, to assist them with this endeavor (131). Grüntzig remembered:

«*Dr. Elias Hanna, a surgical colleague working with Richard Myler in San Francisco, did not have this fear. Dr. Hanna indicated that his bypass grafts would ‘always be better than what was accomplished with dilatation.’*» (86)

**Figure 12:**
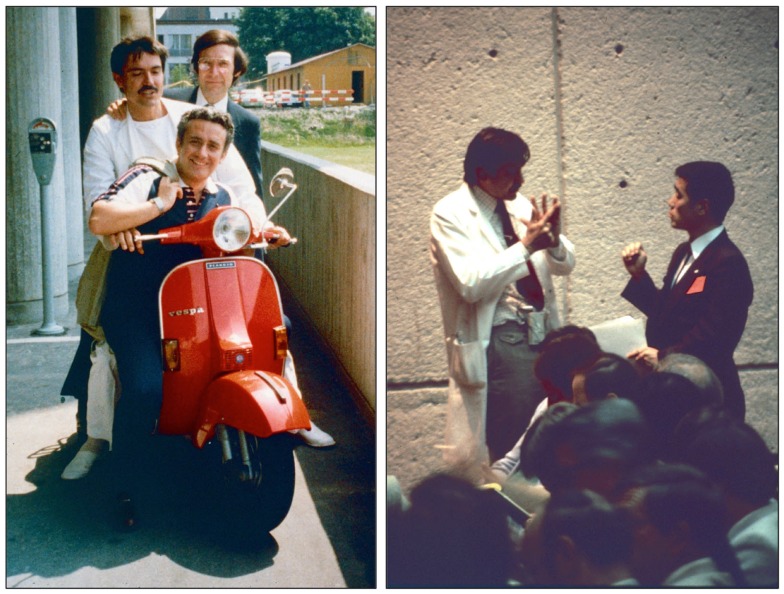
**Left:** Andreas Grüntzig on his Italian Piaggio Vespa scooter in Zürich. Sitting in the front is Richard K. Myler, M.D. with whom Andreas Grüntzig performed the first coronary angioplasties intra-operatively during coronary bypass surgery in May of 1977 in San Francisco. Grüntzig kept this photograph framed on his office desk at Emory University until his death. Shown standing in the back is Simon H. Stertzer, M.D. **Right:** Andreas Grüntzig during discussion with a course attendee of one of the angioplasty demonstration courses held at Emory University, Atlanta, GA, USA. Photographs reproduced with permission of Ernst Schneider, M.D., Zürich.

On May 9, 1977, Grüntzig, with the assistance of Myler performed the first of a number of coronary balloon angioplasties in anesthetized patients during coronary bypass surgery (28, 133, 134) by retrograde insertion into a stenosed coronary artery after arteriotomy. Several coronary balloon angioplasties were done from the opened thorax, and all dilations were done before the surgeons established the aorto-coronary bypass (135). Myler also visited Grüntzig in Zürich (Figure 12) and became a close friend (136–138).

Four months after the first intra-operative coronary angioplasties in San Francisco, and only because Grüntzig had Senning’s unreserved support (36, 38, 39), Grüntzig successfully performed the first PTCA in Zürich in a conscious patient – 38-year old Adolf Bachmann – who had been originally scheduled for bypass surgery because of an isolated LAD stenosis [reviewed in Ref. (28, 131, 133, 135, 139–142)]. The nurse assisting Grüntzig took his notes during the procedure. In his notes, the coronary artery balloon dilatation is referred to as “Coronar-Dotter” (Figure 11) and the balloon device as “*Coronar-Dotter Kath* [eter] *nach Grüntzig*” [*Coronary Dotter catheter according to Grüntzig*], paying tribute to Dotter who had opened this field more than a decade earlier (Figure 11). Indeed, others (and Grüntzig himself) referred to the balloon catheter as the “Grüntzig balloon catheter” (139, 143–146), which subsequently was also utilized for intra-operative transluminal coronary angioplasty (72, 147, 148), for dilating recoarctation of the aorta (149), and for non-cardiovascular applications such as dilation of bronchial stenoses (150, 151), or biliary (152) or ileocolonic strictures (153). Interestingly, the idea of applying “angioplasty” to re-open obstructed nasolacrimal ducts had been introduced already in the 1940 (154), and is now done using Grüntzig balloon catheters (155–158).

Grüntzig published the results of coronary balloon angioplasty as a preliminary report in the Lancet in 1978 (124). He published the results of a larger number of patient cases together with Senning a year later (75).

The success and acceptance of coronary balloon angioplasty in the field was by no means immediate. Grüntzig had only been able to perform the first coronary balloon angioplasty because he had the full support of Senning, who during a conference meeting at the hospital in Zürich with surgeons and internists attending cleared the way for Grüntzig with his famous quote (36, 100, 101):

«*Mister Grüntzig: Do it, if something happens, I will operate*»

[«*Herr Grüntzig: Machen Sie es, falls etwas passiert, operiere ich!*»].

As a cardiac surgeon, Senning was well aware of the potential risks that might be associated with the very delicate procedure in small-lumen arteries. By April 1979, Grüntzig had treated 60 patients applying PTCA, 6 of whom had to undergo emergency bypass surgery to prevent a major myocardial infarction (some of them requiring reanimation) (29, 159); a small percentage of these patients experienced a myocardial infarction; however, according to Turina all these patients survived the PTCA or the surgical procedure. By April 1979, Grüntzig reported a primary success of PTCA in 41 out of 60 patients; in 8 of these patients Grüntzig performed balloon angioplasty in stenosed aorto-coronary bypass grafts (29): at that time, PTCA had been successfully applied by Richard K. Myler, M.D. in San Francisco (43 patients), Simon H. Stertzer, M.D. in New York City (43 patients), and Martin Kaltenbach, M.D., in Frankfurt (16 patients) (29).

Grüntzig’s home institution continued to be hesitant to let him proceed with the PTCA procedure. The skepticism and resistance toward Grüntzig’s new approach expressed by his superiors in Zürich, Siegenthaler and Krayenbühl (38), is also reflected by the fact that Grüntzig had to wait for more than 2 months to have a second patient referred for this procedure after his first successful PTCA (122). As a consequence, 65 out of the 169 patients who underwent PTCA in Zürich came from abroad [the 8th PTCA patient was an American (160)]. In fact, even for the initial report of the first PTCA cases of published in the *Lancet* in February 1978 (161), two of the five reported PTCA procedures had to be done by Grüntzig at the University of Frankfurt (122) with the assistance of Martin Kaltenbach, M.D. (born 1928). Among the first 12 cases, PTCA was initially successful/possible in only 8 of them (39).

In the 1960s, Swiss cardiologist Paul R. Lichtlen, M.D. (1929–2005) spent a fellowship with Richard S. Ross, M.D. (born 1924) at Johns Hopkins University in Baltimore who was introduced to coronary angiography by F. Mason Sones, M.D. at the Cleveland Clinic in the 1950s (162–164). Lichtlen helped to establish coronary angiography in Zürich and subsequently in Europe. In 1972, Grüntzig together with Lichtlen published an article in which the diagnostic sensitivity of a patient questionnaire was compared with coronary angiography as the reference standard for diagnosing coronary artery disease (4). Lichtlen moved to Hannover Medical School in 1973.

Though Lichtlen had recommended the SNSF to fund Grüntzig’s grant application to further develop the clinical development of PTCA (39), he remained cautious about the PTCA procedure. Grüntzig’s first poster on experimental coronary angioplasty (124) presented at the 49th Scientific Sessions of the American Heart Association in Miami in November 1976 had caught the eye of Lichtlen who mentioned it to Emory cardiologist, Spencer King, M.D.:

*You must see the exhibit by this man from Zurich in the next aisle*. (135, 165).

On February 8, 1978, 4 days after Grüntzig’s report of the first five PTCA patients cases had appeared in the Lancet (161), the Zürich Newspaper “Tages-Anzeiger” published a front-page article, showing a photo of Grüntzig and Siegenthaler with the headline *“Zürich’s important contribution to fighting myocardial infarction”* [*“Zürichs wichtiger Beitrag zur Bekämpfung des Herzinfarkts”*] (166). The article was followed by a press conference that was broadcasted by the Swiss national television (SRF) (28). Less than a week after the press conference and after also local German newspapers had praised the new procedure as hope for heart disease patients, on February 13, 1978, Lichtlen wrote a personal letter to Siegenthaler, with copy to Senning and Grüntzig (39):

«*…I consider your approach of informing the public at the present state of this technique with only 10 treated cases und unknown long-term results as very premature and therefore of most questionable value. Hopes which currently by no means can be fulfilled were created among many patients …*»

[*“… Ich halte dieses Vorgehen der Information der Öffentlichkeit im jetzigen Stadium dieser Technik bei lediglich 10 behandelten Fällen und noch völlige unbekannten Lanzeitresultaten für stark verfrüht und damit von sehr zweifelhaften Wert. Es wurden dadurch bei vielen Patienten Hoffnungen erweckt, die in keiner Weise zur Zeit zu erfüllen sind…”*] (39).

Fortunately, time would tell that Lichtlen’s concerns were not justified.

## Andreas Grüntzig: Clinical Teacher and Educator in Vascular Medicine

**Figure 13:**
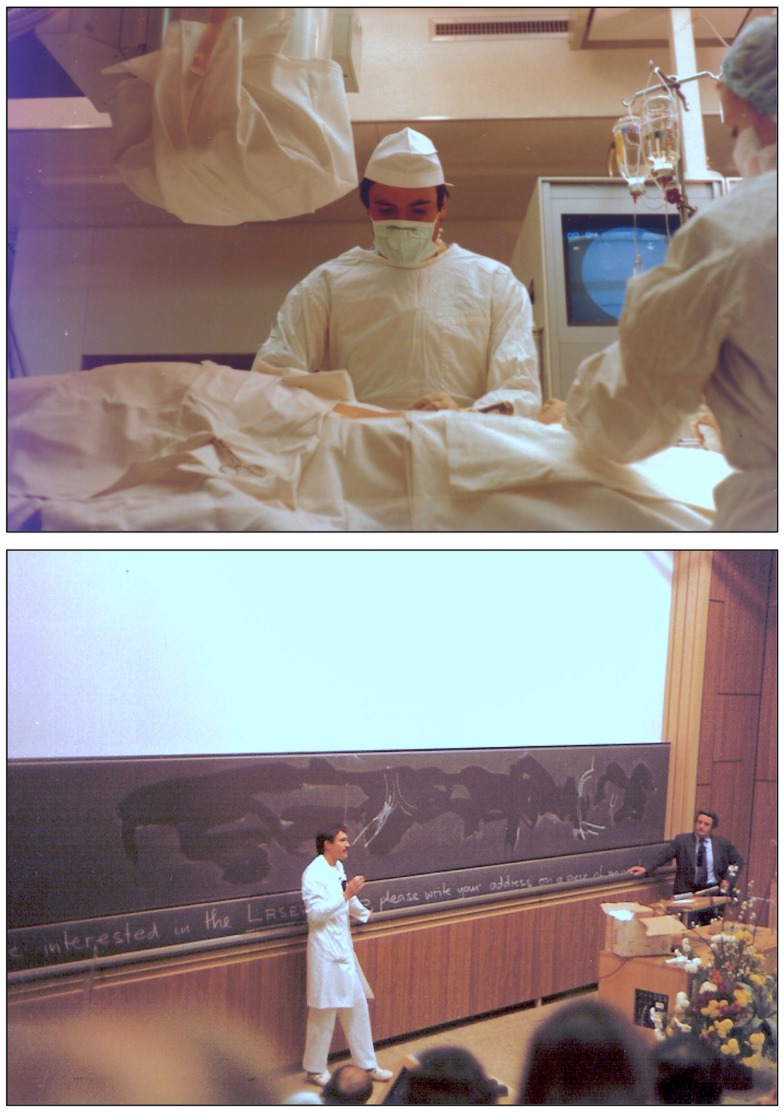
Photographs of Andreas Grüntzig in the late 1970 during the balloon angioplasty courses at the University of Zürich. **Top:** Grüntzig in the catheterization laboratory of the Medical Policlinic during a PTCA procedure at the Kantonspital, which was transmitted live into the lecture hall. **Bottom:** Grüntzig discussing with course attendees in the lecture hall (Grosser Hörsaal Nord, Figure 15) during the 1980 balloon angioplasty course. Shown standing next to blackboard is Richard K. Myler, M.D. Photographs reproduced with permission of Maria Schlumpf, Zürich.

**Figure 14:**
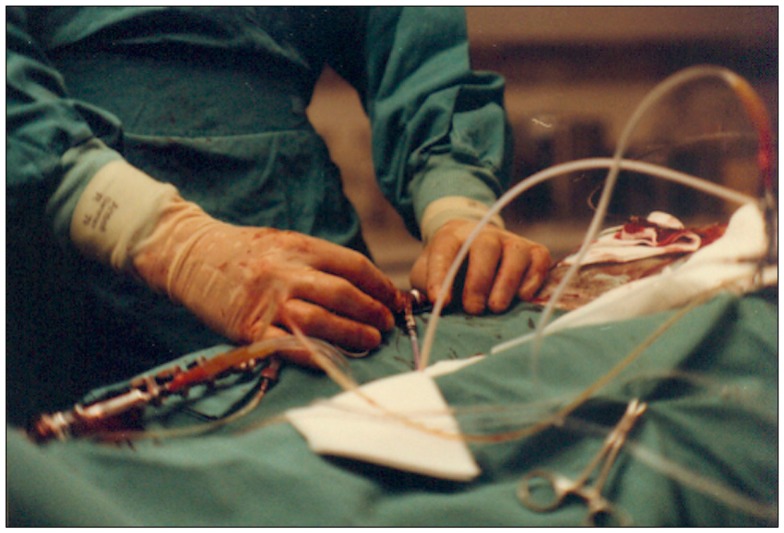
**Photograph of the hands of Andreas Grüntzig during a PTCA procedure in the catheterization laboratory during the 1979 angioplasty course held in Zürich**. The procedure was transmitted live and shown on screens located in the lecture hall shown in Figure 15. Photograph reproduced with permission of Maria Schlumpf, Zürich.

**Figure 15:**
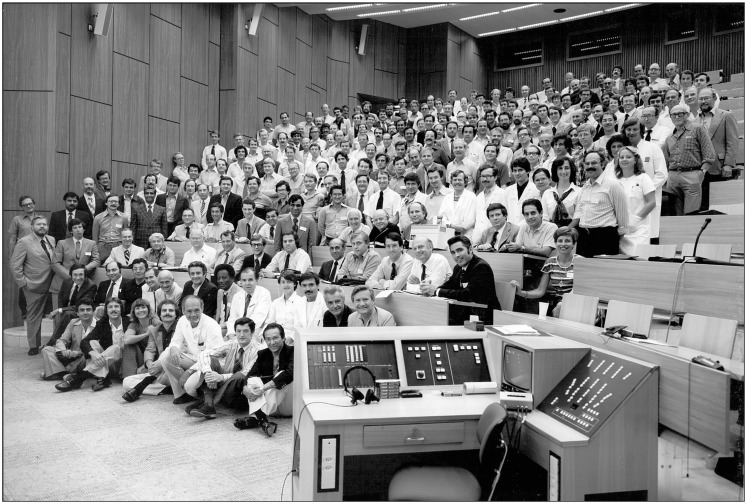
**Photograph of attendees of the last angioplasty demonstration course held at the Grosser Hörsaal Nord at the Kantonspital of the University of Zürich in August 1980**. Most of the pioneers of transluminal and coronary angiography and angioplasty were present, including Charles Dotter, M.D. (second row, 8th from right), Andreas Grüntzig, M.D. (second row, 3rd from right), Melvin P. Judkins, M.D. (third row, 9th from right), and F. Mason Sones, M.D. (fourth row, 4th from right). Also shown are Maria Schlumpf (second row, 4th from right), Richard K. Myler, M.D. (second row, 7th from right), Simon H. Sterzer, M.D. (second row, 12th from right), Grüntzig’s mother, Charlotte (fifth row, 3rd from right), as well as Wilhelm Rutishauser, M.D. (third row, 8th from right) and Hans-Peter Krayenbühl, M.D. (second row, 5th from right), who directed the cardiology division at the Medical Policlinic between 1969 and 1980. Photograph reproduced with permission of Maria Schlumpf, Zürich.

Following the success of the PTCA, cardiology and vascular medicine colleagues came to Zurich hoping that Grüntzig would teach them the new method. These teaching requests became so time-consuming that he could no longer accomplish his daily clinical routine as full-time attending physician at the cardiology division of the Medical Policlinic. Grüntzig therefore decided to start live demonstration courses at the Zurich Kantonsspital performing balloon angioplasty with patients in the catheterization laboratory (28, 36, 133) (Figures 13 and 14). Grüntzig held four live demonstration courses in Zürich between 1978 and 1980 (167), all attended by cardiologists and vascular physicians from around the world. During the first course held on August 7–10, 1978, and with 28 physicians attending, Grüntzig performed 7 PTCAs, 2 femoral PTAs, and 1 renal artery PTA (39). While the first two courses (the second counted 97 attendees) were held in the historical building of the hospital, the third and fourth course accommodating 171 and 221 attendees, respectively, were held in the newly built, large lecture hall (Grosser Hörsaal Nord, Figure 13 bottom and Figure 15). Grüntzig transmitted live from the catheterization laboratory discussing with the audience while being with the patient who was undergoing the angioplasty procedure. Ironically, according to Grüntzig, it was during these live demonstration courses that he learned the most from the comments and questions of his peers (36, 39). Grüntzig enjoyed direct discussion with course attendees during breaks from the angioplasty procedures that he did in the catheterization laboratory (Figure 13), and was considered an extraordinarily gifted teacher by his peers (76, 138, 165, 167).

Although the PTCA method had just been introduced into clinical practice, Grüntzig did not consider this a problem and, according to his former assistant Maria Schlumpf, he was convinced of its safety when performed correctly (36). In fact, Grüntzig was always and first and foremost concerned with the safety of the patient, and initially thought – perhaps because 1 out of 10 patients underwent emergency surgical revascularization in the early years (159) – that only 5–10% of all coronary artery disease patients might be eligible to be treated with his new method. Grüntzig summarized his experience from teaching in the early PTCA courses as follows:

«*But it was the first course in Zürich with the critical audience observing the performance in the very early state of my learning curve which took the most. When I was asked why I chose to perform in that way at the early stage, my answer was: ‘If the method is good, it should work even in a live demonstration. If not, let’s look at it before we endanger our patients!’. It was this type of teaching which directly confronted a critical as well as an enthusiastic audience with the truth and which helped a great deal in teaching and spelling out the method*» (39)

Grüntzig repeatedly emphasized that avoiding complications and ensuring patient safety has utmost priority when selecting patients for coronary balloon angioplasty.

## 1980: Leaving Zürich

His home institution and his superiors were unwilling to provide Grüntzig with the needed support despite the overwhelming success of his new treatment (36, 119, 165). Former colleagues of Grüntzig at the Kantonspital in Zürich described the atmosphere at the time unlike that in the United States:

«*The US environment showed a different attitude to young doctors* […] *In Zürich, you were nobody unless you were a Professor and it was very hierarchical*.» (168).

Siegenthaler, Physician-in-Chief of the Medical Policlinic went by the name of «little Napoleon» [«*kleiner Napoleon»*] among his staff (169), in reference to Napoléon Bonaparte (1769–1821), who has been described as the early nineteenth century dictator of France (170). When confronted with the situation, Siegenthaler blamed the Government of the Cantone of Zürich (Zürcher Regierungsrat) and denied any responsibility on his part, while Grüntzig kept complaining to him that patients on waiting lists for the new treatment were dying (37). Finally, Grüntzig also personally approached Peter Wiederkehr, member of the Zürcher Regierungsrat, who also was unwilling to help (37). Only in 2009, shortly before Siegenthaler’s death (and more than 30 years after Grüntzig’s first successful PTCA at the University of Zürich) Siegenthaler publically admitted that he was afraid at the time that he might have gone to prison should Grüntzig’s procedure had failed (166). In an interview, which he gave in the U.S. in the early 1980s, Grüntzig stated:

«*The only problem I had was a lack of beds in which to put the patients, which caused a long waiting list for PTCA. Patients on the waiting list were having their stenoses close completely, getting MIs, and dying*. […] *I was allowed to treat only two patients a week in Zurich. When I came to Emory, I was able to do two PTCA procedures a day*.» (171).

Grüntzig began looking for a position in Germany at the University of Tübingen, at the University of Frankfurt, and at the University of Düsseldorf, but without success. However, institutions in the United States indicated that they would provide him with the needed support that would allow him to further refine and advance the PTCA method and to treat larger numbers of patients. Several institutions, among them Harvard University and the Cleveland Clinic, offered Grüntzig positions and he finally accepted an offer from Emory University in Atlanta, GA, USA (131, 135). Unlike Switzerland, the United States immediately realized Grüntzig’s capacity and potential to advance cardiovascular medicine. Grüntzig was classified as a “national treasure” by the authorities in 1980 (172); however, he was never granted United States citizenship (96). Emory University had just received a donation of 105 million USD from the Coca-Cola Foundation (an amount which in 2014 would equal approximately 250 million USD), one of the biggest research grants ever given to an academic institution (99), which allowed the hospital to expand on treatment of coronary artery disease using balloon angioplasty technology. Grüntzig accepted a Professorship at Emory University Medical School, with the understanding to serve as the Director of Interventional Cardiovascular Medicine at Emory (119). After his death, to honor and acknowledge Grüntzig’s contributions and pioneering work in the field, Emory University established the «Andreas Gruentzig Cardiovascular Center» to further build upon Dr. Gruentzig’s progress in the area of interventional cardiology (96, 173).

Twenty-five years after Grüntzig had died and only shortly before his own death, Siegenthaler for the first time publically claimed that “Grüntzig had lost sight of his boundaries,” that he had become “rich and world-famous” in Atlanta, GA, USA, and spread the idle rumor that fame and success had gotten to Grüntzig’s head (166). At the same time, Siegenthaler continued to credit and portrait himself as the scientific midwife of balloon angioplasty in books, articles, and on internet websites. Notably, Siegenthaler listed Grüntzig’s article on coronary angioplasty published in the *New England Journal of Medicine* (75) among his own most important publications («Die wichtigsten Publikationen Walter Siegenthalers») (166).

At the last live demonstration course in Zürich in early August of 1980, Dotter received a ring from Grüntzig as a token of appreciation for his achievements in the field of cardiovascular medicine (Figure 15). Upon his return to Oregon – Grüntzig had already decided to leave the University of Zürich – Dotter, on August 22, 1980, in a personal letter (39) wrote to Grüntzig:

«*You proved to be one of the most skilled catheterizers I have ever seen; not only that but a superb teacher and a doctor who never forgot his patient. We will be happy to have you in this country*. […]. *In view of your accomplishments and competency it seems inconceivable that Zürich has not given you a full professorship with tenure. Our gain will be their loss.»* Dotter continues: «*I will admit that in the past I have been at times jealous, especially when I heard people talking about the Grüntzig procedure. I find no jealousy now, only admiration at the thorough and innovative job you have done»* […] *«The ring is on my desk and I will continue to prize it. In an effort to give you something, I am forwarding you a print of our 1968 film on transluminal angioplasty. It’s long out of date, but has certain historical significance*.

*Consider this a sentimental gift from me*.[…]

*Sein echtiger Freund* [sic],

*Charles T. Dotter*»

Today, Dotter’s sentimental gift to Grüntzig is publicly available (73).

At Emory, Grüntzig had only 5 years left to continue teaching and educating fellows and medical colleagues and continued the angioplasty demonstration courses which he had started in Zürich. Between 1980 and 1985, Grüntzig organized 10 courses for his colleagues who came to Emory from all over the world to receive his advice (167) (Figures 9 and 12). Spencer B. King, M.D., remembers Grüntzig’s qualities:

«*Andreas was an incredibly bright and intense guy and very committed to what he was doing, but at the same time he was always open and encouraging of others. That’s what all of his courses that he initially began were about — to share the knowledge. He was active in all aspects of life — work, play, anything. His motto was that if you really wanted something, you should pay the price for it and not worry. He would not have been very good at economizing on anything. He drove fast, he lived fast, and he accomplished a lot in a short period of time*.» (174)

In less than 5 years at Emory, Grüntzig performed more than 3,000 PTCA procedures, “without losing a single patient” (175); his professionalism as a clinician is also evident from an episode told by Spencer B. King, M.D., which happened during one of the angioplasty demonstration courses held at Emory:

«*One day during the lunch break of the course, Andreas was leading a group of people who asked him if he would show them the cath labs. At the same time John Douglas was doing a case on a very obese woman. During the procedure, the woman had fibrillated and John defibrillated her. She literally bounced off the table and onto the floor where John was kneeling, carefully keeping the catheter in place. At that moment, Andreas opened the door and came into the lab with about 10 people trailing behind him. Within a second he assessed the situation and, before anyone else could see, he quickly turned the group around, saying ‘Nothing happening here,’ and ushered everyone out*.» (174)

**Figure 16:**
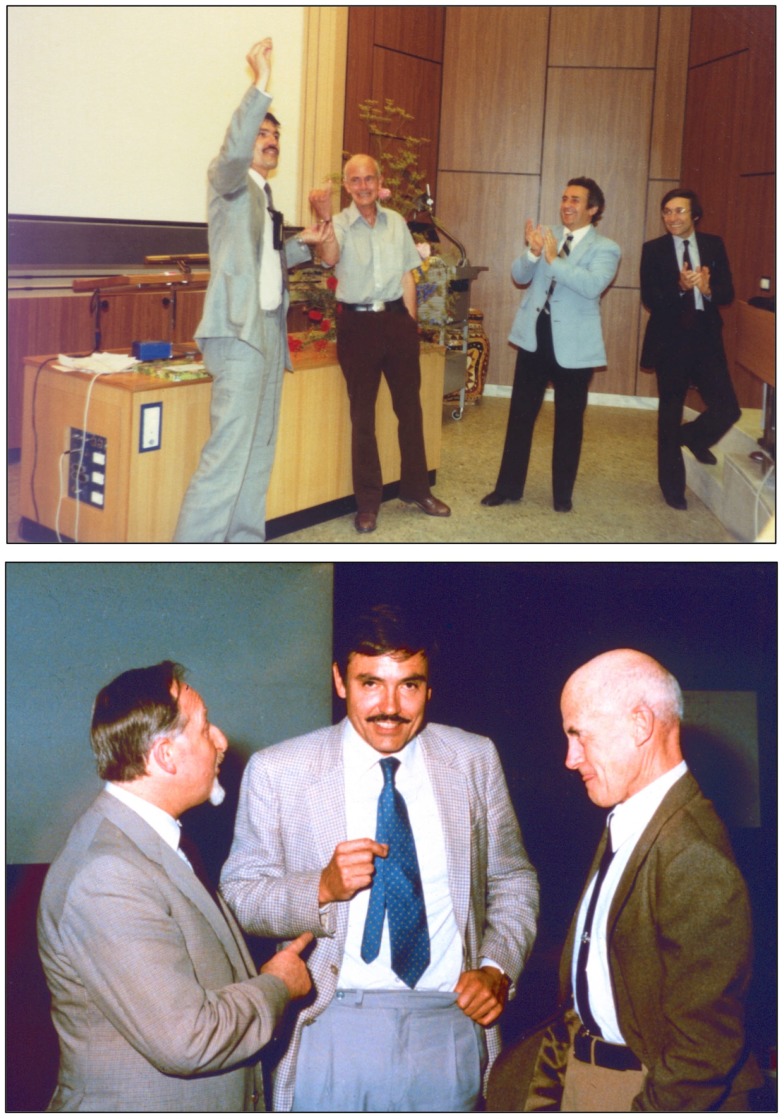
**Top:** Andreas Grüntzig, M.D. presenting Charles T. Dotter, M.D. with a ring as a token of appreciation for his work in the field of interventional vascular medicine. Shown applauding are Richard K. Myler, M.D. and Simon H. Sterzer, M.D. (right). **Bottom:** Three vascular medicine pioneers and founders of endovascular therapy (from left to right): Eberhard Zeitler, M.D. (1930–2011), Andreas Grüntzig, M.D. (1939–1985), and Charles Dotter, M.D. (1920–1985), discussing at a symposium held in 1982 in Nürnberg, Germany. Top photograph reproduced with permission of Maria Schlumpf, Zürich, bottom photograph reproduced with permission of Ernst Schneider, M.D., Zürich.

While at Emory, Grüntzig continued meeting with Zeitler and Dotter in Europe (Figure 16). Only a few months before Grüntzig’s death, Milt Freudenheim, a journalist with *The New York Times*, outlined the financial expectations associated with the predicted growth of coronary angioplasty in cardiovascular medicine; he also described how Grüntzig’s intellectual property of the balloon catheter concept could not be secured by the company who first manufactured the Grüntzig balloon catheter in Switzerland and was soon sold to a larger company (175).

Shortly thereafter, Grüntzig gave his last interview at Emory with Burt Cohen [available online (176)] where he also expresses concerns about the safe future use of his invention:

«*It is easy to be a hero and do a lot of dilatations and a lot of stenoses, but you also then have to be a hero to face the family in which you feel that probably the approach wasn’t right and should have waited a day instead of getting in and trying to elegantly do everything in one session and running into trouble which could have been avoided. So if you want to be a hero so you better be it also in the follow-up and also when you face the patient’s family after you had a trouble*.» (176)

Andreas Grüntzig and his second wife, Margaret-Ann Grüntzig, M.D. (née Thornton), unexpectedly died when his airplane crashed at 3:33 p.m. on October 27, 1985, in Southeastern Monroe county in Forsyth, GA, USA (28, 177). The investigation of the crash that immediately followed Grüntzig’s tragic and early death has not been conclusive. After 30 years, several questions remain unresolved to this day (28, 177).

## Transluminal Balloon Angioplasty: 40 Years On

**Figure 17:**
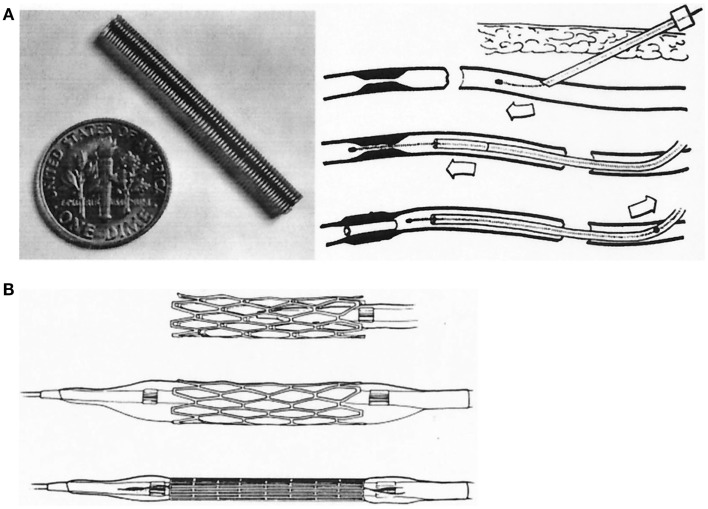
**(A)** Examples of coil-spring, endarterial tube graft, and its percutaneous placement as introduced by Charles T. Dotter, M.D., in 1969. **(B)** Balloon-expandable stents developed by Julio Palmaz, M.D. **(A)** reproduced from Ref. (178) and **(B)** reproduced from Ref. (183), with permission of the publishers.

Building on Dotter’s initial idea that was based on an accidental observation (63, 64, 70), the use of balloon catheters introduced by Grüntzig to restore blood flow in stenosed peripheral and coronary arteries has developed into one of the most successful therapeutic interventions of all time. As demonstrated by Carrel in animal experiments a century ago (15), it was again Dotter who already in 1964 developed the concept of endovascular «splints» and who proposed to use endovascular stents as non-biological structures to improve the stability of the diseased arterial wall (60, 178) (Figure 17). Dotter, Rösch, and colleagues were also the first to use a refined mode of application after balloon angioplasty had been invented and introduced the word “stent” to vascular medicine and for the treatment of peripheral arteries (179, 180). The first coronary stent, initially designated “coronary endo-prosthesis,” was deployed by Jacques Puel, M.D. (1949–2008) at the University Hospital in Toulouse, France, on March 26, 1986 (180, 181), the second one by Ulrich Sigwart, M.D. in Lausanne on June 12, 1986 (182). The field experienced an enormous development, in which Cesare Gianturco, M.D. (1905–1995), Gary S. Roubin, M.D. Ph.D., Julio Palmaz, M.D., and Richard A. Schatz, M.D. (Figure 17) also made important contributions (182–185).

Forty years after its introduction, balloon angioplasty continues to save lives of patients with acute myocardial infarction (131, 135, 140, 142). Balloon angioplasty saved patients from disability by preventing leg amputations, as demonstrated by both Dotter and Grüntzig with their very first patients (22, 60). The discovery of balloon angioplasty represents perhaps one of the best early examples of translational medicine in recent medical history (26, 140). What Dotter as early as 1964 in his first article (60) and 1968 in his film (73) envisioned to be applied in patients with coronary artery disease some day, Grüntzig translated into clinical application within a little more than a decade (26, 75, 161).

Coinciding with the 40^th^ anniversary of the first balloon angioplasty in peripheral artery disease at the University of Zürich, the U.S. FDA approved the first drug-coated balloon for peripheral vascular interventions on October 12, 2014 (186), and corresponding results were recently published by Jaff and associates (187). Future applications of balloon angioplasty in humans may also include the combination of adjuvant drug treatment targeting disease mediators (6, 188).

In 2013, Grüntzig was posthumously awarded the «*TCT Career Achievement Award*» for founding the field of interventional cardiology (189). Although Grüntzig foresaw the developments of balloon catheter application in many ways (in the 1980s, he expressed his interest in performing PTAs of extracranial arteries – which has become clinical practice today), Grüntzig’s clinical interests were by no means limited to interventional cardiology. In fact, he had received extensive training in public health and disease prevention and in the last years of his life also developed a keen interest in preventive cardiology, a field, which also would see major developments in recent decades (6, 190).

## Andreas Grüntzig: The Human Being

On June 25, 2014, Andreas Grüntzig would have celebrated his 75th birthday. It is most obvious that in his professional life as a physician and as a scientist Grüntzig – in many ways – succeeded in achieving more in half a lifetime than most of us ever will. He did persevere against all obstacles that he was confronted with, both in Germany and in Switzerland, with enormous will power, optimism, intellect, hard work, and after all – remaining the same person, “a real Mensch” that he had been since the early days. J. Willis Hurst, M.D. (1920–2011), a close friend and colleague of Grüntzig, remembered in 1986:

«*What a role model he was. The greatest stimulus to learning is the behavior of another person who exhibits noble attributes. Everyone who knew him realized he was unique. As each individual discovered his great attributes, he or she became a better person. This, I believe, was his greatest teaching achievement*.» (167).

Grüntzig’s personality also becomes apparent in his response when he learned about the death of a close friend and colleague, Pierre Wirz, M.D. (1935–1982). Grüntzig proclaimed: *“God always calls his most beloved ones first!”* [*Der Herrgott holt sich seine Lieblinge zuerst!*] (39). Wirz, who trained Grüntzig in coronary angiography, and Pierre Levis, M.D., another close friend of Grüntzig (Figure 8), started doing PTA procedures in peripheral arteries using the Grüntzig balloon catheters at Triemli City Hospital in Zürich in February of 1975. Thus, Levis, who is still active doing balloon angioplasties, will soon also have a 40^th^ angioplasty anniversary to celebrate and thus has the longest experience of using Grüntzig balloon catheters of anyone in the world today. Spencer B. King, M.D., in an anecdote, reflects on Grüntzig’s personality:

«*Sure he was a hard negotiator and demanding of others as he was of himself, but when he perceived that others would be hurt, he was very sensitive. Once while looking for office space, I obtained a convenient suite of rooms for him near the cath lab. Everything was set, but when he discovered the feelings of the people who would be displaced, he refused the space. Dr. Hurst came forward with alternative accommodations*» (165)

The formed head of the Division of Thoracic and Cardiovascular Surgery at Heinrich-Heine Universität in Düsseldorf between 1972 and 1990, Wolfgang Bircks, M.D. (born 1927), remembers visiting Andreas Grüntzig at Emory University Hospital in Atlanta shortly before his death and recalls an extraordinary kindness in the way Grüntzig treated his coworkers in the catheterization laboratory. When Bircks met Andreas Grüntzig’s brother Johannes after Grüntzig had died in the airplane crash, he offered him his condolences and commented on his death with the following words (191):

«*Andreas Grüntzig made his exit like he used to make his entrance: with a thunderbolt*»

[«*Andreas Grüntzig ist so abgetreten, wie er aufzutreten pflegte: mit einem Donnerschlag*!»]

Wolfgang Bircks, M.D.

## Competing interests and financial relationships

The authors declare that they have no commercial or financial relationships or other interests related to the work presented that could be construed as potential competing interests.
